# A comparison of massively parallel nucleotide sequencing with oligonucleotide microarrays for global transcription profiling

**DOI:** 10.1186/1471-2164-11-282

**Published:** 2010-05-05

**Authors:** James R Bradford, Yvonne Hey, Tim Yates, Yaoyong Li, Stuart D Pepper, Crispin J Miller

**Affiliations:** 1 Applied Computational Biology and Bioinformatics, Cancer Research UK, Paterson Institute for Cancer Research, The University of Manchester, Wilmslow Road, Manchester M20 4BX UK; 2 Molecular Biology Core Facility, Cancer Research UK, Paterson Institute for Cancer Research, The University of Manchester, Wilmslow Road, Manchester M20 4BX UK

## Abstract

**Background:**

RNA-Seq exploits the rapid generation of gigabases of sequence data by Massively Parallel Nucleotide Sequencing, allowing for the mapping and digital quantification of whole transcriptomes. Whilst previous comparisons between RNA-Seq and microarrays have been performed at the level of gene expression, in this study we adopt a more fine-grained approach. Using RNA samples from a normal human breast epithelial cell line (MCF-10a) and a breast cancer cell line (MCF-7), we present a comprehensive comparison between RNA-Seq data generated on the Applied Biosystems SOLiD platform and data from Affymetrix Exon 1.0ST arrays. The use of Exon arrays makes it possible to assess the performance of RNA-Seq in two key areas: detection of expression at the granularity of individual exons, and discovery of transcription outside annotated loci.

**Results:**

We found a high degree of correspondence between the two platforms in terms of exon-level fold changes and detection. For example, over 80% of exons detected as expressed in RNA-Seq were also detected on the Exon array, and 91% of exons flagged as changing from Absent to Present on at least one platform had fold-changes in the same direction. The greatest detection correspondence was seen when the read count threshold at which to flag exons Absent in the SOLiD data was set to *t*<1 suggesting that the background error rate is extremely low in RNA-Seq. We also found RNA-Seq more sensitive to detecting differentially expressed exons than the Exon array, reflecting the wider dynamic range achievable on the SOLiD platform. In addition, we find significant evidence of novel protein coding regions outside known exons, 93% of which map to Exon array probesets, and are able to infer the presence of thousands of novel transcripts through the detection of previously unreported exon-exon junctions.

**Conclusions:**

By focusing on exon-level expression, we present the most fine-grained comparison between RNA-Seq and microarrays to date. Overall, our study demonstrates that data from a SOLiD RNA-Seq experiment are sufficient to generate results comparable to those produced from Affymetrix Exon arrays, even using only a single replicate from each platform, and when presented with a large genome.

## Background

### RNA-Seq technology

Massively Parallel Nucleotide Sequencing (MPNS) allows the rapid generation of gigabases of sequence data at a relatively low cost per residue. A variety of platforms exist, but all rely on the generation of a large number of relatively short sequences, known as 'tags' or 'reads' that can then be aligned to a target database, or assembled *de novo *into contiguous sequences. In many MPNS experiments, it is possible to treat the set of reads generated during a sequencing run as an unbiased sampling of the total nucleotide complement of the cells, making it possible to use the number of reads aligning to a given locus as an estimate of its abundance. A major application that depends on this is RNA-Seq [1-7]. Here, the proportion of reads matching a given transcript is used as a measure of its expression level.

Unlike hybridization-based techniques such as qPCR or microarrays, RNA-Seq does not rely on pre-determined probes designed against known target sequences, allowing it to be used to search for novel transcription at previously uncharacterized loci. Although this can be achieved successfully using tiling arrays, microarrays can suffer from binding affinity constraints that make it difficult to design reliable probes targeted at certain sequences, rendering parts of the genome inaccessible [8]. In addition, recent research has revealed extensive amounts of alternative splicing in the human genome [9], leading to the prediction that there are many novel transcripts arising from uncharacterized splicing events, and/or the incorporation of additional exons up- and downstream of a given gene. By seeking reads that cross exon-exon boundaries, MPNS can be used to identify novel arrangements of exons, and thus, novel transcripts [10].

Although powerful, RNA-Seq is not without challenges, and many of the computational caveats that apply to microarray analysis are equally applicable, including an inability to distinguish between loci with 100% sequence similarity, and a dependence on appropriate algorithms, statistics and annotation tools to support the data analysis [11,12]. Critical to the approach is the need to generate sufficient reads to cover each locus at sufficient depth to give reliable estimates of expression. This can be significantly more than might be expected because the approach relies on random sampling of the fragmented transcriptome. The wide dynamic range of transcription data means that a relatively small number of highly expressed loci can account for the majority of the reads in the study (in the data that follows, for example, 50% of the exonic reads map to less than 1% of exons in MCF-10a).

### Affymetrix Human Exon 1.0ST arrays

Affymetrix Exon arrays are currently the most dense arrays designed specifically for profiling gene expression [13]. They feature approximately 1.2 million probesets that aim to target every known and predicted exon in the entire genome, supporting the detection of alternative splicing events [14,15]. Each probeset consists of up to four probes targeting a defined probe selection region (PSR). A PSR can correspond to an exon supported either by RefSeq mRNA evidence, Expressed Sequence Tag (EST) evidence or purely computational predictions. Approximately 50% of probesets target loci outside Ensembl-defined protein coding exons, allowing Exon arrays to detect transcription outside well-characterized loci [16]. In this respect, they share some of the potential advantages of RNA-Sequencing platforms.

### Objectives

Several recent studies have compared gene expression microarrays with RNA-Seq using both human [6,17] and mouse [5,7,18] samples, reporting good correspondence between gene expression and fold changes, and higher gene detection rates in RNA-Seq than on an array. In this study, we adopt a more fine-grained approach by comparing RNA-Seq data from an Applied Biosystems (AB) SOLiD v3 platform to exon-level microarray data produced using Affymetrix Human Exon 1.0ST arrays. Unlike the previous studies above, the use of Exon arrays makes it possible to assess the performance of RNA-Seq in two key areas: detection of expression at the granularity of individual exons, and discovery of transcription outside annotated loci. In addition, we leverage the Exon array data to assess the level of technical background present in our RNA-Seq dataset, the influence of reads that map to multiple genomic loci, and to define the fold change threshold used to call differentially expressed exons. As such, we do not treat the Exon arrays as a gold standard but simply as a reliable source of an unknown and independent set of true positives with which to compare RNA-Seq.

## Results and Discussion

### Datasets

RNA from two cell lines, MCF-7, a breast cancer line, and MCF-10a, a normal epithelial line, was used in this study. One sample from MCF-10a and two technical replicates from MCF-7 (labelled MCF-7_r1 and MCF-7_r2) were analysed on the SOLiD platform (see Methods), and the same RNA samples hybridised in triplicate to Affymetrix Exon 1.0ST arrays, as previously described [19]. MCF-7 is an abnormal and heterogeneous cell-line. Consequently, sequence differences between its genome and that of the reference used to design the arrays may result in some probes failing to hybridise to their target sequence. However, both MCF-10a and MCF-7 achieve similar probeset detection rates. These are significantly above the QC thresholds recommended by the manufacturer, and well within the bounds that are typical for cell line microarray data. A low detection rate, which would be indicative of a substantial number of probesets failing to hybridize successfully (as would be expected if genetic instability was a significant confounding factor), was not observed. Furthermore, similar effects would also cause issues with the RNA-Seq data, since SNPs and polymorphisms will result in increased error rates during alignment. Therefore, it is unlikely that the genomic complexity of MCF-7 would have a significant effect on these data.

Variability between RNA-Seq replicates was low; high correspondence was observed at both the exon (*r *= 0.87; Additional File 1: Supplementary Figure S1A) and gene expression levels (*r *= 0.92; Additional File 1: Supplementary Figure S1B). For cross platform correspondence, the Exon array replicate from each cell line with the largest proportion of detected probesets was selected, together with the RNA-Seq MCF-7 replicate having the highest number of reads (MCF-7_r1).

**Figure 1:**
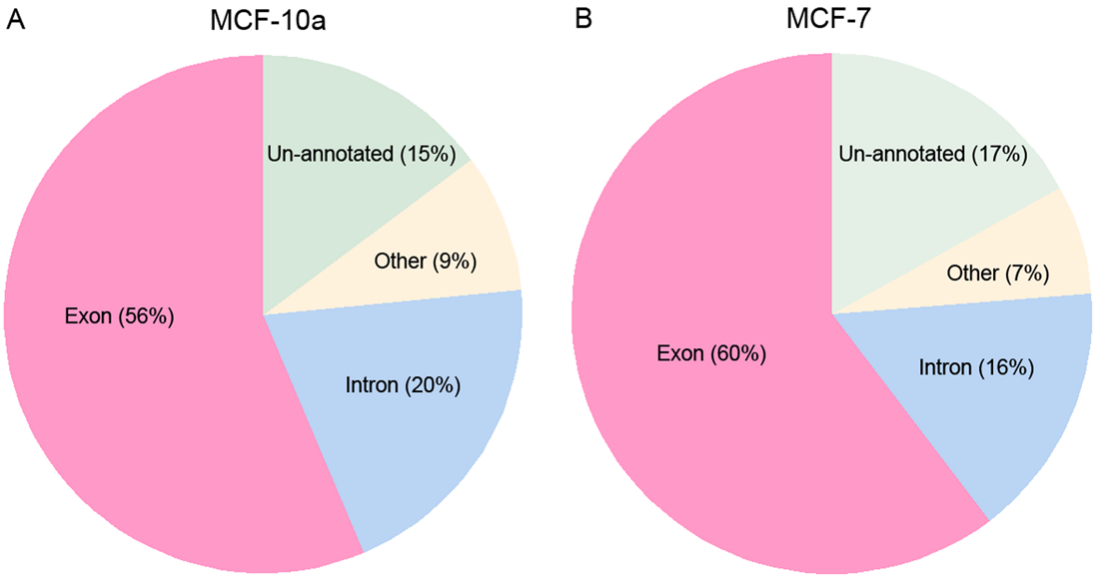
**Read locations**. The proportion of unique reads in (A) MCF-10a and (B) MCF-7, mapping to four genomic locations: known exons and introns, as defined by Ensembl, other annotated regions including ESTs, Genscan predictions and Exon array probe selection regions, and un-annotated regions.

50 base reads were aligned to NCBI build 36 of the human genome allowing 6 mismatches per read. Unless otherwise stated, reads that matched to multiple loci were removed. Table 1 summarizes the read counts obtained from each of the sequencing runs. A total of 28,371,318 reads from MCF-10a and 28,882,179 from MCF-7_r1 mapped uniquely to the genome. 77% (21,709,397) of these mapped to an Ensembl known transcript [20] in MCF-10a and 76% (22,031,344) in MCF-7_r1, and of these 74% (15,996,190) and 79% (17,439,762) respectively mapped to a known exon (Figure 1). Overall, 85% (24,159,893) of reads in MCF-10a, and 83% (23,988,482) in MCF-7_r1 mapped to annotated loci that included known transcripts, Ensembl Genscan predictions, ESTs, and sequences between the 5'- and 3'-most probes within each probeset. Over half of all known exons in both cell lines (58%; 168,678/291,229 in MCF-10a, and 59%; 170,721/291,229 in MCF-7) featured at least one matching read.

**Table 1:** Summary of read counts across different genomic locations.

	MCF-10a	MCF-7_r1	MCF-7_r2
Total	286,197,907	302,129,896	150,762,975
After error filtering	173,966,873	205,050,087	113,512,672
Mappable	47,524,622	46,330,340	33,697,119
Uniquely mappable	28,371,318	28,882,179	22,223,910
Location			
Ensembl known			
Total	21,709,397	22,031,344	16,980,001
Exon	15,996,190	17,439,762	12,800,399
Intron	5,713,207	4,591,582	4,179,602
All annotation^1^	24,037,188	23,854,633	18,830,788
Exon Junctions			
Known	1,010,785	1,225,448	-
Putative	16,548	23,540	-

^1^Known, predicted and EST transcripts

### Determination of background

Estimates of transcript abundance on Exon arrays can be affected by biases due to cross hybridisation as a result of probes hybridising to off-target transcripts [8,21]. Likewise, RNA-Seq can suffer from reads that align to incorrect loci due to sequencing errors, or differences between the reference sequence and that of the genome under study. In this work, we define these reads as "background". In order to quantify background in our RNA-Seq data, we carried out two analyses. First we considered genes on the Y chromosome, and secondly, we compared exon detection on the SOLiD platform with Present/Absent calls on the Exon Array.

### Expression levels on the Y chromosome

Due to its absence in female samples, no RNA should originate from the Y chromosome in MCF-10a and MCF-7; any reads uniquely mapping here should therefore be due to technical artefacts. We therefore measured the proportion of exons targeted by at least one read in both MCF-10a and MCF-7. To account for pseudo-autosomal regions where the probability of mapping a read unambiguously is reduced, we only considered exons whose length exceeded 100 bases after subtracting the number of non-unique loci (see Methods) found within that exon. This reduced the total number of exons under consideration by 36% from 1835 to 1178. Only three (0.25%) of these exons on the Y chromosome in MCF-10a and six (0.51%) exons in MCF-7 were targeted by at least one mappable read. Four of these exons from both cell lines were mapped to by more than one read, all of which derived from two genes (*USP9Y *and *NLGN4Y*) with high sequence identity to corresponding genes (*USP9X *and *NLGN4X*) on the X chromosome. Supplementary Figure S2 (Additional File 1) shows that the number of exons expressed on the Y chromosome was significantly lower than on all other chromosomes. For example, on the X chromosome, which had the second lowest proportion of expressed exons, over 50% of exons in both MCF-10a and MCF-7 were targeted by at least one mappable read.

### Exploiting Present/Absent calls on the Exon array

On Exon arrays, probesets with signals separable from background can be flagged Present (P), or Absent (A) using the Detection Above Background (DABG) score [22], which estimates the probability that a probeset's signal is similar to the general background distribution. The DABG score is calculated by comparing each probe to a GC-content matched reference pool of background probes, and combining these data across the probeset to yield a final *p*-value. Any probeset with a *p*-value below a pre-defined threshold is flagged Present.

**Figure 2:**
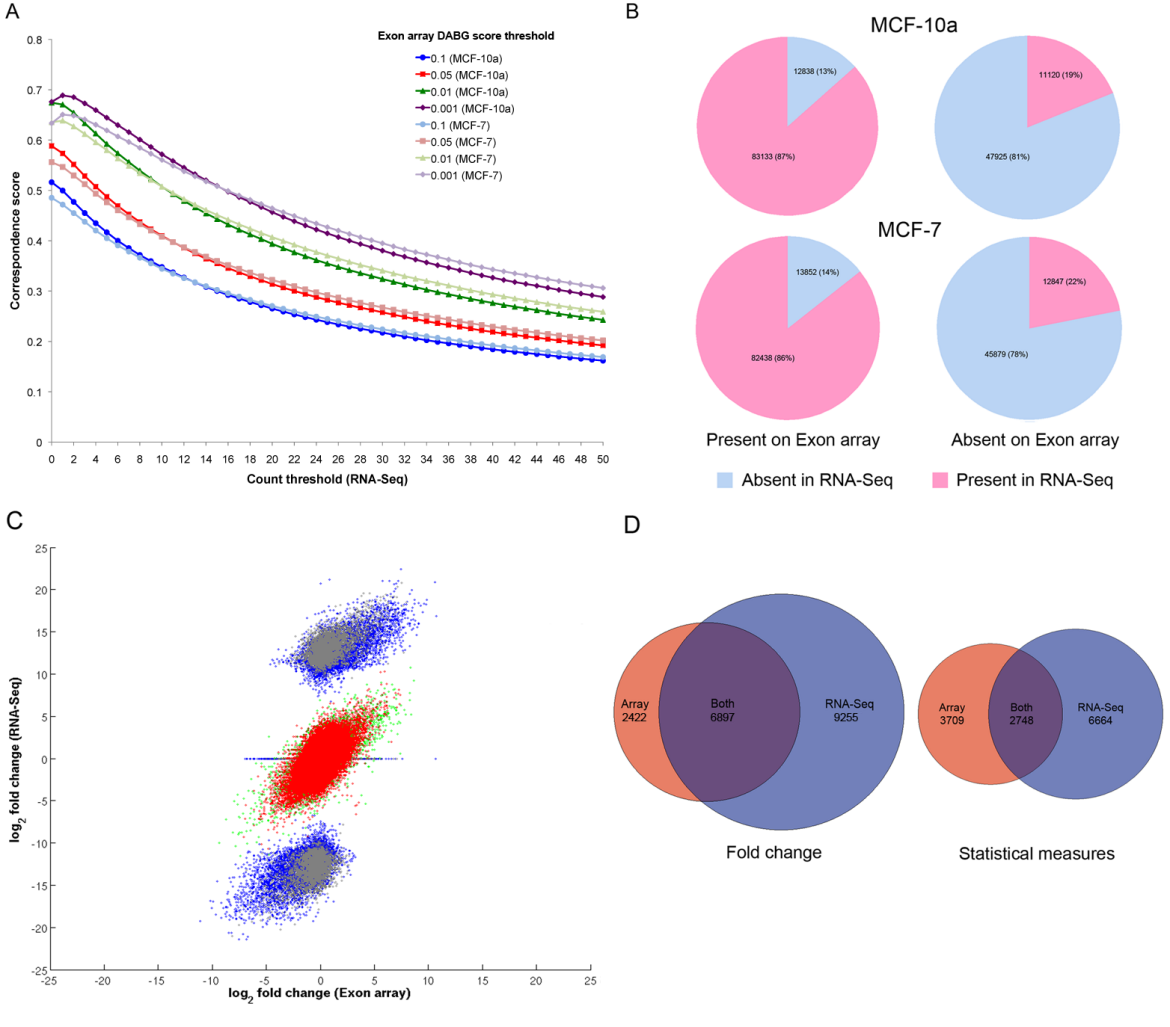
**Correspondence between RNA-Seq and Exon arrays**. (A) Determination of the read count threshold giving optimum correspondence between both platforms with respect to Present/Absent calls. (B) Present/Absent call correspondence at a read count threshold of zero in RNA-Seq and a DABG score threshold of 0.01 on the array. (C) Comparison of fold changes between RNA-Seq and the array. Red dots indicate exons flagged as Present (P) in both samples and on both platforms (PP->PP). Grey dots indicate exons flagged as Absent (A) in at least one sample on both platforms (AA->AA, PA->PA, AP->AP, PA-AP, AP->PA, AA->PA, AA->AP, PA->AA, AP->AA). Note that, due to the density of the data, some grey points representing exons Absent in both RNA-Seq samples (zero fold change) are masked by other colours. Blue dots indicate exons Absent in at least one RNA-Seq sample but flagged Present in both array samples (PA->PP, AA->PP, AP->PP), and green dots represent exons Present in both samples in RNA-Seq but flagged Absent in at least one sample on the array (PP->PA, PP->AA, PP->AP). (D) Overlap between numbers of exons called differentially expressed by the array and RNA-Seq using (Left) a log_2 _fold change threshold of 2.0 on the array and 3.0 in RNA-Seq (left) and a LIMMA *p*-value threshold of 1 × 10^-4 ^on the array and an Audic-Claverie *p*-value threshold of 1 × 10^-7 ^in RNA-Seq (right). These thresholds lead to the greatest equivalence between platforms using an overlap metric based on the *CS *(Equation 2).

In order to further assess background level in the RNA-Seq data, we compared P/A calls for the 155,016 exons targeted by a single probeset on the Exon array with P/A calls for the same exons on the SOLiD platform, where an exon was defined as Present if the number of reads mapping to it was greater than a threshold *t*. Since such a comparison is dependent on the detection threshold used for each platform, we took an unbiased approach in which read count and DABG thresholds were varied independently (Figure 2A). At each combination of expression level and DABG threshold, we calculated a correspondence score (*CS*; see Methods). We initially considered three DABG *p*-value cut-offs of 0.1, 0.05 and 0.01 and found that *CS *was maximal (0.67) at a read count greater than zero and DABG threshold of 0.01 in MCF-10a (Figure 2A). For MCF-7, significant correspondence (*CS *= 0.64) was also seen at these thresholds. At this read count and DABG threshold in MCF-10a, 87% (83,133) of the exons called Present on the Exon array were also called Present using the RNA-Seq data, and 81% (47,925) of the exons called Absent on the array were also flagged Absent on SOLiD (Figure 2B). We observed similar results in MCF-7, suggesting that the genetic instability of this cell line had little impact on detection rates in both RNA-Seq and on the array: 86% (82,438) of exons present on the Exon array were Present on SOLiD, and 78% (45,879) of exons Absent on the Exon array were also Absent on SOLiD. Furthermore, exons detected solely by RNA-Seq tended to have a low level of expression in both cell lines (Additional File 1: Supplementary Figures S3A and S3B).

We also considered a fourth, more stringent DABG *p*-value cut-off of 0.001 at which detection correspondence was generally higher than at the other DABG thresholds (Figure 2A). However, at a read count threshold of zero, CS increased only marginally by 0.002 in MCF-10a from a DABG cut-off of 0.01 to 0.001, and decreased in MCF-7, thus failing to compensate for the significant reduction of probesets called Present on the Exon array (14,833 in MCF-10a and 16,049 in MCF-7). We also observed that the maximum CS was achieved in both cell lines at a read count threshold of one. This result was expected since the majority (58%; 5424/9301) of exons in MCF-10a with a single mappable read were called Absent on the array at this DABG cut-off, compared with only 42% (3885/9301) at a cut-off of 0.01, thus the read count threshold achieving maximal CS increases to account for this.

In addition, we also performed the same analysis with normalised expression values calculated using Equation 2. Supplementary Figure S4 (Additional File 1) shows similar trends to Figure 2A with the optimal correspondence (with a DABG cut-off of 0.01) occurring at a normalised expression value of 0.3.

Taken together with the low number of reads mapping to the Y chromosome, these data suggest that the background error rate is extremely low in RNA-Seq data. We therefore chose *t *< 1 as the read count threshold at which to flag exons Absent in the SOLiD data. In addition, our results confirm that a DABG cut-off at 0.01 is a sensible choice to call probesets Present/Absent on the Exon array.

### Expression level and fold change correspondence

Given the differences between the platforms and the protocols used to prepare their samples, it is perhaps unreasonable to expect significant correlation between their raw expression levels, although we did see some correlation (*r *= 0.55, MCF-10a; *r *= 0.53, MCF-7) for those exons flagged Present on both platforms (Additional File 1: Supplementary Figures S5A and S5B respectively). However, fold changes should be consistent across platforms, particularly for features that have sufficient signal to noise ratio to yield a reliable fold-change in both arms of the study. Fold changes between the log_2 _exon expression levels from MCF-7 and MCF-10a were calculated independently for the Exon array and SOLiD platform, and then compared (Figure 2C). Note that, to avoid taking logs of zero (a situation that arises in exons without a mappable read), we added a small constant (0.0001) to all normalised expression levels calculated using Equation 2. This results in the appearance of three clusters of data in Figure 2C: the uppermost cluster in which the majority of data are a result of Absent (zero count) to Present fold changes in RNA-Seq, the lowermost cluster resulting from Present to Absent fold changes, and a middle cluster that includes both Present-Present and Absent-Absent fold changes. Fold changes showed good correspondence (*r *= 0.59) between platforms when data points featuring at least one Absent-flagged sample were removed (i.e. after eliminating comparisons involving data points with poor signal-noise ratio, essentially all data except the red points of Figure 2C). The majority (91%; 7077/7757) of exons flagged as changing from Absent to Present on at least one platform had fold-changes in the same direction, but with different magnitudes. This is to be expected since Absent-flagged exons are likely to have poor signal to noise ratio, and thus unstable ratios. Those flagged Absent in both cell lines in at least one platform showed least correspondence.

### Effect of removing multiple-targeting reads

In these analyses, we removed reads that target multiple loci. A consequence of this is that paralogous genes with regions of 100% sequence identity greater than or equal to the read length may be under-counted, since reads will not be reported at these sites. Since this might result in loss of sensitivity for these exons on the RNA-Seq platform, we repeated the above analysis using both unique and multi-targeting reads. All locations targeted by a read were considered, for example, if a read targeted two different transcripts then the read counts of both transcripts would increase by one. As expected, the proportion of exons from MCF-10a flagged Present on both SOLiD and the Exon array increased by 2% from 87% (83133) to 89% (85238), but at a cost of 2297/59045 (4%) exons called Present on SOLiD but Absent on the Exon array. A slight reduction in expression level correspondence from *r *= 0.54 to *r *= 0.51 was also seen in MCF-10a. Thus, the indiscriminate addition of reads that target multiple loci results in reduced correspondence between the two platforms. In addition, hits to the Y chromosome also increased with 426/1834 (23%) exons in MCF-10a and 399/1834 (22%) exons in MCF-7 having at least one mappable read.

### Differential expression

A key application of RNA profiling technology is the ability to reliably call differential expression for well-characterized protein coding exons. Unlike microarrays, approaches for identifying differential expression in RNA-Seq data are in their early stages of development, particularly those that can process only one RNA-Seq replicate per condition (a situation that is, at least in the short-term, likely to arise frequently, given the current high cost of the approach). In this study, we chose two measures of differential expression for the RNA-Seq experiment: fold-change and the Poisson-based approach of Audic-Claverie (AC) [23]. For all microarray comparisons, triplicate exon array data were used, and differential expression was found using LIMMA [24,25].

Our goal was to find a combination of thresholds from each differential expression measure that maximises correspondence between the RNA-Seq and Exon array data sets. To do this, we examined the set difference and intersection between the exons called differentially expressed by each platform. The approach assumes that each platform will generate its own independent set of false positives and negatives, and that although these will result in less than perfect correspondence, the intersection, which represents the consensus between both platforms, will be enriched for true positives. We used *CS *(Equation 3) as a metric, as before. Note that by trying to maximise the correspondence between platforms, we are not treating the exon arrays as a gold standard but simply as a reliable source of an unknown and independent set of true positives against which to try to maximise the overlap.

Thresholds were varied independently for each platform, and *CS *calculated (Additional File 1: Supplementary Figures S6A and S6B). The threshold-pair where *CS *was maximal was taken as the point at which to perform comparisons. We found that the highest correspondence between platforms (*CS *= 0.49) occurred with a higher log_2 _fold change threshold in RNA-Seq (3.0) than the arrays (2.0; Additional File 1: Supplementary Figure S6A), suggesting that the SOLiD platform tends to report higher fold-changes. This is to be expected, since the lower background (essentially zero) will result in less fold-change compression (due to the presence of a constant background value in the numerator and denominator of the ratio calculation). The distribution of fold changes on the SOLiD platform versus the Exon array is shown in Supplementary Figure S7 (Additional File 1). At these thresholds, 6897 exons were called differentially expressed on both platforms, 2422 exons were called differentially expressed on the Exon array only, and 9255 exclusively on SOLiD (Figure 2D).

When statistical measures were considered, the greatest correspondence (*CS *= 0.28) was seen at a *p*-value of 1 × 10^-7 ^on SOLiD and 1 × 10^-5 ^on the Exon array (Additional File 1: Supplementary Figure S6B), although given the different methods of deriving the *p*-values it is difficult to compare the AC threshold with LIMMA directly. Nevertheless, at these thresholds, 6664 exons were called differentially expressed in RNA-Seq only, compared to 3709 on the Exon array, with 2748 exons called differentially expressed on both platforms, further suggesting that the SOLiD platform is more sensitive to calling differential expression than the Exon array (Figure 2D). See also Supplementary Figure S8 (Additional File 1), which compares the *p*-values calculated by LIMMA and AC back transformed to quantiles on a normal distribution and signed by the direction of the fold change, making the differences in the tails of the population particularly apparent.

### Length bias with the AC measure

Many of the statistical tests thus far applied to RNA-Seq data suffer from length bias [26]. Length bias is expected under a uniform sampling assumption. Consequently, if differences in transcript length are not adequately accounted for, there will be more power to detect differences in expression for longer transcripts. Simply dividing read counts by exon length does not correct for this [26], and the use of AC would then be inappropriate since the data would no longer be Poisson-distributed. To determine the extent of length bias at the exon level, we binned exons by length and calculated the proportion called differentially expressed in each bin for both the SOLiD and the Exon array data, using the fold change and AC thresholds at which *CS *is maximal determined in the previous section. 11% (9412/82821) of all exons in the RNA-Seq data were called differentially expressed using AC. When data were stratified by length, 22% (1489/6755) of exons in the upper quartile (> 239 bases), and 6% (1326/23750) in the lower quartile (< 119 bases; Additional File 1: Supplementary Figure S9A) were found to be differentially expressed. On the Exon array, 8% (6457/82821) of all exons were called differentially expressed using LIMMA compared to 9% (591/6755) of those in the upper quartile, and 7% (1699/23750) in the lower. Thus, the expected length bias when using AC was observed in the RNA-Seq data but not the Exon array dataset. No length bias was seen when fold change was used to define differential expression (Additional File 1: Supplementary Figure S9B).

### Differential expression using edgeR

Recently, a new differential expression calling algorithm, edgeR [27], has been proposed, which models count data using a negative binomial model that can be regarded as an over-dispersed Poisson model. To calculate differential expression, edgeR requires only a pair of replicates in one condition, allowing its use with the single MCF-10a sample and two MCF-7 replicates of this study. Thus it provides a useful comparison with the AC measure that uses only a single sample from each condition. Like the AC measure, edgeR does not normalise counts for exon length although total read numbers in each sample are considered (Equation 5).

Supplementary Figure S10A (Additional File 1) is analogous to Figure 2C in showing fold change correspondence between RNA-Seq and the Exon array. However, in this case fold changes in RNA-Seq are calculated by edgeR (using both MCF-7 replicates) and on the Exon array by LIMMA (using all three replicates from both cell lines). As expected, with the additional information provided by the replicates, correspondence between fold changes increased significantly from *r *= 0.59 to *r *= 0.71 when data points featuring at least one Absent-flagged exon were removed. As before, we assessed differential expression calls at various combinations of fold change thresholds in RNA-Seq and on the Exon array, and again found the highest correspondence between platforms (*CS *= 0.51) at a log_2 _fold change threshold of 3.0 in RNA-Seq and 2.0 on the arrays (Additional File 1: Supplementary Figure S10B). At these thresholds 6939 exons were called differentially expressed on both platforms, 2555 exons were called differentially expressed on the Exon array only, and 8373 exclusively in RNA-Seq (Additional File 1: Supplementary Figure S10C).

An increase in *p*-value correlation between RNA-Seq and the Exon array was also observed with edgeR (*r *= 0.68; Additional File 1: Supplementary Figure S10D) compared to AC (*r *= 0.56; Additional File 1: Supplementary Figure S8). At a *p*-value threshold of 0.001 on RNA-Seq and 0.00001 on the Exon array, maximal differential call correspondence was reached (*CS *= 0.48; Additional File 1: Supplementary Figure S10E), with 3221 exons called differentially expressed on both platforms, 2762 exons called differentially expressed in RNA-Seq only, and 3308 exons called differentially expressed solely on the Exon array (Additional File 1: Supplementary Figure S10F). Whilst it is difficult make direct comparisons between their *p*-values, the lower *p*-value required to achieve maximal correspondence with edgeR compared to AC suggests that edgeR, used with the parameters given in Methods, is more conservative in its differential expression calls.

As with AC, some bias towards calling differential expression in longer exons was evident. Employing the same procedure as before and using thresholds of 0.001 on RNA-Seq and 0.00001 on the Exon array, 7% (5983/83896) of all exons in the RNA-Seq data were called differentially expressed using edgeR. When data were stratified by length, 13% (865/6709) of exons in the upper quartile (> 239 bases), and 4% (918/23762) in the lower quartile (< 119 bases; Additional File 1: Supplementary Figure S11) were found to be differentially expressed.

### Identification of known and novel splicing events

An advantage of RNA-Seq is the opportunity to characterize splicing events by seeking reads that span exon-exon junctions [6,28]. An additional 1,010,785 and 1,225,448 reads mapped in the sense orientation to our data set of 263,574 known exon-exon junctions in MCF-10a and MCF-7 respectively (only 0.01% of hits mapped in the antisense direction in both cell lines). As a result, 31% of junctions in both MCF-10a (80,756) and MCF-7 (82,708) were confirmed by at least one unique read. The majority (67,558) of junctions seen in MCF-10a were also seen in MCF-7.

Accounting for exon-junction reads increased detection call correspondence between RNA-Seq and Exon arrays across both cell lines. *CS *increased from 0.67 to 0.69 in MCF-10a, and from 0.64 to 0.65 in MCF-7. This equated to an extra 3178 and 3470 exons called present on both SOLiD and the Exon array in MCF-10a and MCF-7 respectively, at the expense of only 1861 and 2076 additional exons called present on SOLiD but absent on the Exon array.

In order to detect novel splice events, searches were performed against a database of 5,172,880 putative exon-exon junctions generated by permuting known exon sequences within each gene (see Methods). A further 16,548 reads in MCF-10a and 23,540 in MCF-7 mapped to this dataset, capturing 11,725 and 16,850 novel junctions respectively (1,795 in both cell lines). The majority of these junctions (9,702 in MCF-10a and 13,613 in MCF-7) were mapped by a single read, which suggests they represent rare splicing events. 72 high confidence novel splice junctions in MCF-10a and 129 in MCF-7 were mapped to by at least five unique reads with more than one start site and overlapping the junction by at least 12 nucleotides. Of these, 25 were found in both cell lines. A list of the high confidence junctions is given as Supplementary Table S1 (Additional File 2).

### Identification of novel loci of transcription

Given that 15% and 17% of reads in MCF-10a and MCF-7 respectively mapped to regions on the genome that are currently un-annotated, we performed a systematic search to identify which of these regions are likely to represent novel loci of transcription, focusing in particular on putative novel exons. To do this, we first grouped reads into clusters by seeking sets of overlapping reads, and then merging adjacent clusters separated by 50 bases. 2,061,888 and 1,840,985 such clusters were found in MCF-10a and MCF-7 respectively. These were then labelled exonic (if at least one of their nucleotides was part of a known exon), intronic, predicted exonic (if overlapping a Genscan predicted or EST exon) or predicted intronic. Those that remained were classed as intergenic.

### Discovery of putative novel exons with RNA-Seq

Given the low levels of background observed in these data, it is clear that the majority of these read clusters, regardless of location, are indicative of real transcription. This concurs with other studies that have found as much as 90% of the human genome to be transcribed [9]. However, not all of these regions will represent protein-coding mRNA. To identify these, we first sought read clusters that had expression levels similar to those of existing exons, and then attempted to find additional functional validation by using Pfam [29] to search these sequences against a database of candidate protein domains.

Using the number of reads contributing to a cluster to indicate the expression level of that locus, we determined an expression level cut-off for calling novel exons. This was achieved by using known exonic and intronic read clusters to define a threshold that maximised the *CS *score in Equation 3. We found that a read count of 19 in MCF-10a and 16 in MCF-7 gave predictions that most closely matched the existing gene annotation (Additional File 1: Supplementary Figure S12). 29,508 and 27,008 read clusters, not previously labelled exonic or predicted exonic, exceeded the thresholds in MCF-10a and MCF-7 respectively. Whilst the majority of these were part of known introns (21,574 in MCF-10a and 19,248 in MCF-7), 4449 (15%) and 4900 (18%) were found in intergenic regions of MCF-10a and MCF-7 respectively. The remainder (3485 in MCF-10a and 2860 in MCF-7) resided in predicted introns.

We chose to focus on the intergenic read clusters for the domain analysis. Prior to this step, we removed read clusters in the bottom quartile (<120 base pairs) of the known exon length distribution, and translated the nucleotide sequence corresponding to each of the remaining read clusters in all three reading frames. Any clusters containing a stop codon were removed. The remaining putative protein sequences (120 from MCF-10a and 173 from MCF-7) were scanned for Pfam domains. A total of 28 read clusters contained either a full or partial Pfam domain: 16 in MCF-10a and 12 in MCF-7, and of these 8 were found at similar loci in both cell lines (Additional File 1: Supplementary Table S2).

### Comparison with Exon arrays

Affymetrix Exon arrays feature many probesets targeting loci with relatively weak evidence for transcription; approximately 50% target outside the core Ensembl exon annotations. We compared the loci predicted from the RNA-Seq data, with the locations of these non-core probesets. Of the 4449 intergenic read clusters in MCF-10a exceeding the read-count cut-off, 2664 (60%) overlapped with a probeset target region extended by 300 nucleotides at either end. Of these probesets, 92% (2462/2664) were called Present. Likewise in MCF-7, 62% (3038/4900) of read clusters were located at or near a probeset selection region, and of these, 91% (2767/3038) were called Present. In general, we found that as the read count of a cluster increased, the probability of finding a neighbouring probeset also increased, and that probeset was more likely to be called Present (Additional File 1: Supplementary Figure S13). When domain filtering is included, 26 of the 28 read clusters (93%) in Supplementary Table S2 (Additional File 1) map to or near probesets, of which 16 were called Present. This compares favourably with the overlap observed for the core annotations.

## Conclusions

The success of a transcript-profiling platform depends on a number of technical constraints, including sensitivity and selectivity, its coverage, and its accuracy. These must be balanced against more pragmatic considerations that include cost, throughput and ease of data-analysis. Unlike array-based technologies, the sensitivity and accuracy of RNA-Seq is highly dependent on having significant reads to cover the genome with enough detail to provide valid data for low-abundance transcripts.

In this study we show that data from a SOLiD RNA-Seq experiment is sufficient to generate results comparable to those produced from Affymetrix Exon arrays, even using only a single replicate from each platform, and when presented with a large genome. In part, this is achievable only because the RNA-Seq protocols used here preserved 'strandedness' information, allowing comparisons to be performed in the knowledge of the 5'-3' orientation of each read. This is likely to be a pre-requisite for many experiments. Furthermore, the consistency of results between both cell lines reinforced our original belief that the genomic complexity of MCF-7 has little impact on the comparison between RNA-Seq and the Exon array. Our main focus was at the exon level, and this, to our knowledge, is the first study to present such a fine-grained comparison between the two platforms. In addition, it was possible to identify a set of intergenic loci expressed at significant levels, the majority (60%) of which match probesets on the Exon array. Further analysis suggested that many of them would result in the expression of known protein-coding domains, if translated, providing evidence that these are not simply transcriptional artefacts. Finally, by searching a dataset of novel exon-exon junction sequences, we were able to identify thousands of putative novel splicing events.

Of course, although the data presented here show significant technical correspondence between platforms, this does not obviate the need for replication at the biological level in order to assess the likelihood that these changes are simply chance occurrences. Indeed, where replicate data is available, this information is useful and can lead to improved performance, as evidenced by the closer correspondence achieved between the RNA-Seq and the array data with edgeR and utilising the two MCF-7 replicates. As technology continues to improve it is reasonable to expect the number of reads generated in a single sequencing run to increase substantially, allowing multiple samples to be processed on a single slide and so reducing the cost of the experiment dramatically. One clear benefit of this would be the opportunity to include an increased number of replicates. As this happens RNA-Seq will become an increasingly cost-effective approach to whole transcriptome profiling.

## Methods

### RNA library preparation

Two cell lines, MCF-7, a breast cancer line, and MCF-10a, a normal epithelial line, were processed according to manufacturer's standard protocols. Total RNA (5 μg) from both cell lines were depleted of 18S and 28S rRNA and 1 μg of each sample were enzymatically fragmented using 1 unit of RNase III (Ambion) and incubating at 37°C for 10 minutes. The fragmented RNA was then size selected using the flashPAGE™ fractionator (Ambion) to collect RNA fragments ranging in size from ~50-150 nucleotides in length. The RNA fragments were then ligated to adaptors, converted into cDNA and amplified by 15 cycles of PCR using the SOLiD™ Small RNA Expression Kit (Ambion). The PCR reactions were purified using the Qiagen Minelute PCR purification kit and separated on a native Novex 6% TBE polyacrylamide gel (Invitrogen) and stained with SYBR gold. PCR products ranging in size from ~150-200 bp (corresponding to RNA fragment insert sizes of ~60-110 nucleotides) were cut out of the gel, the gel slices were shredded and the products eluted overnight and precipitated. The gel-purified material was quantitated by nanodrop and prepared for emulsion PCR and SOLiD sequencing.

### Genome level alignments and annotation

Reads of length 50 bases originating from each sample were first aligned to the human genome (US National Center for Biotechnology Information (NCBI) Build 36.3) using Applied Biosystems' SOLiD™ System Analysis Pipeline Tool (Corona Lite; http://solidsoftwaretools.com/gf/project/corona/). Six mismatches were tolerated as recommended by the Corona documentation, and reads with an expected error rate (Equation 1) greater than 6 were discarded prior to matching.(1)

where *n *is the base position in the read and *p *represents the predicted probability that the colour call at position *n *is incorrect. *p *is calculated from the Quality Value (QV) of the colour call where QV = -10log_10_(*p*). Filtering in this way removes 39% of the reads from MCF-10a, 32% from MCF-7_r1 and 25% from MCF-7_r2, whilst retaining 94%, 94% and 97% of the uniquely mappable reads respectively.

Reads that matched multiple loci were removed from the analysis and the resultant alignment files pre-processed to generate 'pile-ups' against each chromosome. In total, 47,524,622, 46,330,340 and 33,697,119 reads (including those matching at multiple loci) were mapped to the genome in MCF-10a, MCF-7_r1 and MCF7_r2 respectively, and of these, 28,371,318, 28,882,179 and 22,223,910 were uniquely mappable to the genome. Once data were aligned to the genome, the BioConductor package exonmap and associated annotation database, X:Map [16] based on Ensembl version 52, were used to group reads according to the exons, transcripts and genes they mapped to. These groupings were then used to inform subsequent statistical analysis. For cross-platform correspondence, we only considered exons targeted by a single probeset; consequently 155,016 exons were used in the comparison.

### Exon-exon junctions

We anticipated that some of the reads that did not map contiguously to the human genome would align to exon-exon junctions, therefore, additional searches were performed against a dataset of 263,574 known exon-exon junctions as defined by Ensembl version 52. To ensure that a 50 base read mapped to a splice junction, only the last 49 bases of the first exon and the first 49 bases of the second exon were considered (if the exon exceeded length 49). Reads that matched to more than one junction loci, or elsewhere on the genome were discarded, resulting in 1,010,785 and 1,225,448 reads mapping uniquely to exon-exon junctions in MCF-10a and MCF-7_r1 respectively.

In order to detect novel splicing events, we generated a dataset of 5,172,880 novel splice junctions by permuting known exons within each gene. Again, exons of length greater than 49 bases were truncated so the length of each exon pair never exceeded 98 nucleotides. A total of 16,548 and 23,540 reads (that had thus far not been mapped to either the genome or a known exon-exon junction) in MCF-10a and MCF-7 respectively mapped to this novel splice junction dataset. Note that, unless otherwise stated, splice junction reads were not included in detection call correspondence or the expression level calculation (Equation 2).

### Measurement of expression level

We adapted the RPKM measure of [5] to calculate a normalized expression level (*E*) based on the read count across the region of interest (such as an exon):(2)

where *S *is the number of reads mapping to the region, *L *is the region length, *U *is the number of non-unique loci across the exon (see below), *T *corresponds to the total number of uniquely mappable reads in each cell line, and *C *is a constant set to 1 × 10^9 ^in this study. To avoid taking logs of zero, we added a small constant (0.0001) to all normalised expression levels.

### Correspondence Score

To measure Present/Absent correspondence between RNA-Seq and Exon array technologies we used a modified version of the Matthew's Correlation Coefficient [30] to calculate a correspondence score, *CS*:(3)

where *A *indicates the number of exons Present on both platforms, *B *indicates the number of exons Absent on both platforms, *C *indicates the number of exons Present in the RNA-Seq data but Absent on the Exon array, and *D *indicates the number of exons Absent in RNA-Seq but Present on the Exon array. A *CS *of -1 means that all exons called Present in RNA-Seq are called Absent on the Exon array and vice versa, a *CS *of zero means that correspondence is no better than random, and a *CS *of 1 indicates perfect correspondence.

We also use Equation 3 to measure correspondence between sets of exons called differentially expressed in RNA-Seq versus the Exon array at different thresholds. In this case, A indicates the number of exons called differentially expressed on both platforms, B indicates the number of exons called unchanging on both platforms, C represents the number of exons called differentially expressed in the RNA-Seq data but not the Exon array and D represents the number of exons called unchanging in RNA-Seq but differentially expressed on the Exon array.

Likewise, to identify the optimal threshold (*t*) for defining exonic read clusters, we adapted Equation 3 so that A represents the number of exonic read clusters achieving a read count greater than *t*, B indicates the number of intronic read clusters achieving a read count less than *t*, C indicates the number of exonic read clusters achieving a read count less than *t*, and D indicates the number of intronic read clusters achieving a read count greater than *t*.

### Statistical tests for differential expression

#### Audic-Claverie

The statistical test of Audic-Claverie [23] was used to define exon differential expression between the two cell lines. Audic-Claverie is a model based on Poisson statistics, and has been previously applied to SAGE [31] and RNA-Seq [6] expression data. The statistic is calculated according Equation 3.(4)

where *x *indicates the number of reads across an exon in MCF-10a, *y *indicates the number of reads across the corresponding exon in MCF-7, and *N*_1 _and *N*_2 _represent the total numbers of unique reads in MCF-10a and MCF-7 respectively. In this case, the *p*-value indicates the probability of obtaining y counts in MCF-7 given x counts in MCF-10a.

#### edgeR

A recently published Bioconductor [32] package, edgeR [27], was also used to measure exon differential expression between the two cell lines. It models count data as negative binomial (NB) distributed (Equation 5), and employs an empirical Bayes procedure to moderate the degree of over-dispersion across the exons.(5)

for exon *e *and sample *i*, and where *M*_*i *_is the library size, ϕ_*g *_is the dispersion, and *p*_*gj *_is the relative abundance of exon *e *in experimental group *j *to which sample *i *belongs.

For our purposes, we used tag-wise dispersion with the smoothing parameter, prior.n, set to 10. Library sizes were set to the total number of reads in each sample.

### Non-unique loci identification

There are a significant number of positions where, for a given 50-base sequence, one or more identical 50-mers are found elsewhere on the genome. At these loci, the probability of finding a uniquely-mappable read given our six mis-match tolerance is lower than at other positions, possibly preventing the detection of expression at these sites. We therefore performed an exhaustive all-against-all search for all non-unique 50-mers in the genome and recorded where they matched against the reference. In detail, starting from each base position on a chromosome we took a 50 base region of consecutive sequence and searched for an identical 50 base match elsewhere on the genome. If one or more matches were found then that base position and all start positions of the matches were marked with a "1", otherwise the base position was marked with "0". In this way, a profile of ones (corresponding to non-unique loci) and zeros (unique-loci) was generated for each chromosome. 6% of genomic loci were defined as non-unique using these criteria.

## Abbreviations

AC: Audic-Claverie; CS: Correspondence Score; DABG: Detection Above Background; MPNS: Massively Parallel Nucleotide Sequencing; PSR: Probe Selection Region.

## Authors' contributions

JB performed the statistical analyses and drafted the manuscript. YH performed the RNA-Seq and Exon array experiments. TY and YL participated in the statistical analyses. SP participated in the conception and design of the study. CM conceived of the study, and participated in its design and coordination and helped to draft the manuscript. All authors read and approved the final manuscript.

## Supplementary Material

Additional file 1 **Supplementary Material**. Supplementary Text ("Pooling RNA-Seq replicates", "Gene level correspondence"), Supplementary Figures S1-S14, and Supplementary Table S2. Click here for file

Additional file 2 **Supplementary Table S1**. A list of novel exon junctions detected in (a) MCF-10a and (b) MCF-7. Click here for file
