# Supplementary material for: A comparison of massively parallel nucleotide sequencing with oligonucleotide microarrays for global transcription profiling
Source: BMC Genomics. 2010 May 5;11:282. doi: 10.1186/1471-2164-11-282 (PMC2877694; doi:10.1186/1471-2164-11-282)
Supplement: Additional file 1 — Supplementary Material. Supplementary Text ("Pooling RNA-Seq replicates", "Gene level correspondence"), Supplementary Figures S1-S14, and Supplementary Table S2. [file 1471-2164-11-282-S1.DOC]

A comparison of massively parallel nucleotide sequencing with oligonucleotide microarrays for global transcription profiling

^1^James R. Bradford, ^2^Yvonne Hey, ^1^Tim Yates, ^1^Yaoyong Li, ^2^Stuart D. Pepper, ^1^Crispin J. Miller

^1^Applied Computational Biology and Bioinformatics, Cancer Research UK, Paterson Institute for Cancer Research, The University of Manchester, Wilmslow Road, Manchester M20 4BX UK.

^2^Molecular Biology Core Facility, Cancer Research UK, Paterson Institute for Cancer Research, The University of Manchester, Wilmslow Road, Manchester M20 4BX UK.

Supplementary Text

Pooling RNA-Seq replicates

A major issue with RNA-Seq is the number of mappable reads required for accurate determination of expression, since this can affect pooling and bar-coding strategies, and thus the overall cost of an experiment. In order to investigate this, we pooled the MCF-7_r1 and MCF-7_r2 replicates to give 51,106,089 uniquely mappable reads, and repeated the above comparisons to exon arrays. An additional 3467 exons that were previously flagged Absent in the RNA-Seq data but Present on the arrays were detected as Present in the combined RNA-Seq dataset, and a further 3793 exons were called Present in RNA-Seq but Absent on the Exon Array. Thus, the number of exons detected in the RNA-Seq data increased, as expected. However, the increase in the number of exons called Present in RNA-Seq but Absent on the Exon Array resulted in the overall correspondence in detection between platforms falling slightly from *CS*=0.64 to *CS*=0.62. By contrast, correspondence in raw estimates of exon abundance and fold change both increased slightly (from *r*=0.52 to *r*=0.54, and from *r*=0.59 to *r*=0.64, respectively, *p*<1x10^-17^ in both cases). Inspection of the MCF-7_r2 replicate revealed a higher fold change correspondence (*r*=0.63) than MCF-7_r1 (*r*=0.59), and similar exon expression level correspondence despite 6,658,269 less reads, suggesting that some of these gains may be due to factors other than sequencing depth.

Gene level correspondence

The main focus in this work was at the exon level. However, the majority of previous studies have compared RNA-Seq to microarrays at the gene level. For this reason, we present results of the gene level comparison between RNA-Seq and Exon arrays. Note that for measuring gene expression, we only counted reads mapping to exons comprising the gene.

As expected, we observed higher correspondence at the gene level than at the exon level, most likely due to the potential for larger sample sizes across genes than across exons. A correlation in expression levels of *r*=0.75 in MCF-10a and *r*=0.73 in MCF-7 was achieved between 11,144 genes with at least one exon flagged Present in RNA-Seq, and one probeset called Present on the Exon array (Supplementary Figures S14A and S14B respectively). These results compared to *r*=0.54 (MCF-10a) and *r*=0.52 (MCF-7) at the exon level. Fold change correspondence (*r*=0.71; Supplementary Figure S14C) between these same genes was also higher than that at the exon level (*r*=0.59). Overall, these results are in good agreement with previous studies [1, 2].

To measure differential expression between genes in RNA-Seq, we used both fold change and a statistical test. Similar to the exon level, maximal correspondence (*CS*=0.53) was seen at a fold change of 4.5 on SOLiD and 3.0 on the Exon array. At these thresholds 75 genes were called differentially expressed by both platforms, and 102 genes by SOLiD only and 34 exclusively on the Exon array (Supplementary S14D). The statistical test involved a two-sided Wilcoxon Rank-Sum test of the null hypothesis that expression levels across each exon in a gene from MCF-10a and those from the corresponding gene in MCF-7 are from identical continuous distributions with equal medians, against the alternative that they do not have equal medians. On the Exon array, we used the same statistical test but on individual probeset intensities across the gene’s exons. Exons with zero counts or probesets called Absent were ignored. Maximal correspondence (*CS*=0.44) was seen at a *p*-value threshold of 0.00001 on both platforms, and at this level 86 genes were called differentially expressed on both platforms, with 149 and 67 genes called exclusively on SOLiD and the Exon array respectively (Supplementary Figure S14D).

Supplementary Tables

Supplementary Table S2: A list of 28 highly expressed intergenic read clusters with evidence of a Pfam domain [3].

| Cell line | Chr | Strand | Start | Stop | Read count | Probeset | Present probeset | Pfam acc | Pfam ID |
| --- | --- | --- | --- | --- | --- | --- | --- | --- | --- |
| MCF-10a | 2 | + | 165138487 | 165138705 | 85 | Yes | Yes | PF01399 | PCI |
| MCF-7 | 2 | + | 165138519 | 165138705 | 171 | Yes | Yes | PF01399 | PCI |
| MCF-7 | 2 | + | 182267571 | 182267744 | 21 | Yes | No | PF06487 | SAP18 |
| MCF-10a | 2 | + | 234148378 | 234148636 | 88 | Yes | Yes | PF00443 | UCH |
| MCF-7 | 3 | - | 32651738 | 32651914 | 56 | Yes | Yes | PF01248 | Ribosomal_L7Ae |
| MCF-10a | 3 | - | 101866632 | 101866901 | 46 | Yes | No | PF00241 | Cofilin_ADF |
| MCF-7 | 3 | - | 101866633 | 101866901 | 105 | Yes | No | PF00241 | Cofilin_ADF |
| MCF-10a | 3 | + | 143652027 | 143652205 | 28 | Yes | Yes | PF00069 | Pkinase |
| MCF-10a | 5 | + | 37121059 | 37121267 | 20 | Yes | Yes | PF00900 | Ribosomal_S4e |
| MCF-10a | 5 | - | 108258026 | 108258185 | 35 | Yes | Yes | PF01423 | LSM |
| MCF-7 | 5 | - | 108258034 | 108258191 | 38 | Yes | Yes | PF01423 | LSM |
| MCF-7 | 5 | + | 153853909 | 153854160 | 28 | Yes | Yes | PF07200 | Mod_r |
| MCF-10a | 5 | + | 153854168 | 153854314 | 33 | Yes | No | PF07200 | Mod_r |
| MCF-10a | 7 | - | 23570179 | 23570324 | 56 | Yes | No | PF00037 | Fer4 |
| MCF-7 | 7 | - | 23570184 | 23570337 | 22 | Yes | No | PF00037 | Fer4 |
| MCF-10a | 7 | - | 63007383 | 63007828 | 92 | Yes | Yes | PF04981 | NMD3 |
| MCF-7 | 7 | - | 142720015 | 142720205 | 70 | Yes | Yes | PF01230 | HIT |
| MCF-10a | 7 | - | 142720016 | 142720205 | 110 | Yes | Yes | PF01230 | HIT |
| MCF-7 | 8 | + | 57663623 | 57663781 | 41 | Yes | No | PF01907 | Ribosomal_L37e |
| MCF-10a | 8 | + | 57663624 | 57663782 | 23 | Yes | No | PF01907 | Ribosomal_L37e |
| MCF-10a | 11 | - | 90488024 | 90488345 | 25 | Yes | No | PF01237 | Oxysterol_BP |
| MCF-10a | 12 | + | 62502417 | 62502604 | 94 | Yes | Yes | PF00076 | RRM_1 |
| MCF-7 | 12 | + | 62502418 | 62502605 | 145 | Yes | Yes | PF00076 | RRM_1 |
| MCF-7 | 12 | + | 93508817 | 93508997 | 46 | Yes | No | PF01423 | LSM |
| MCF-7 | 16 | - | 2653604 | 2653905 | 18 | No | No | PF00665 | rve |
| MCF-10a | 17 | + | 30356321 | 30356775 | 23 | Yes | Yes | PF10219 | DUF2055 |
| MCF-10a | 19 | - | 41510304 | 41510493 | 27 | No | No | PF02093 | Gag_p30 |
| MCF-10a | 19 | - | 62520523 | 62520737 | 26 | Yes | Yes | PF05903 | DUF862 |

Supplementary Figures


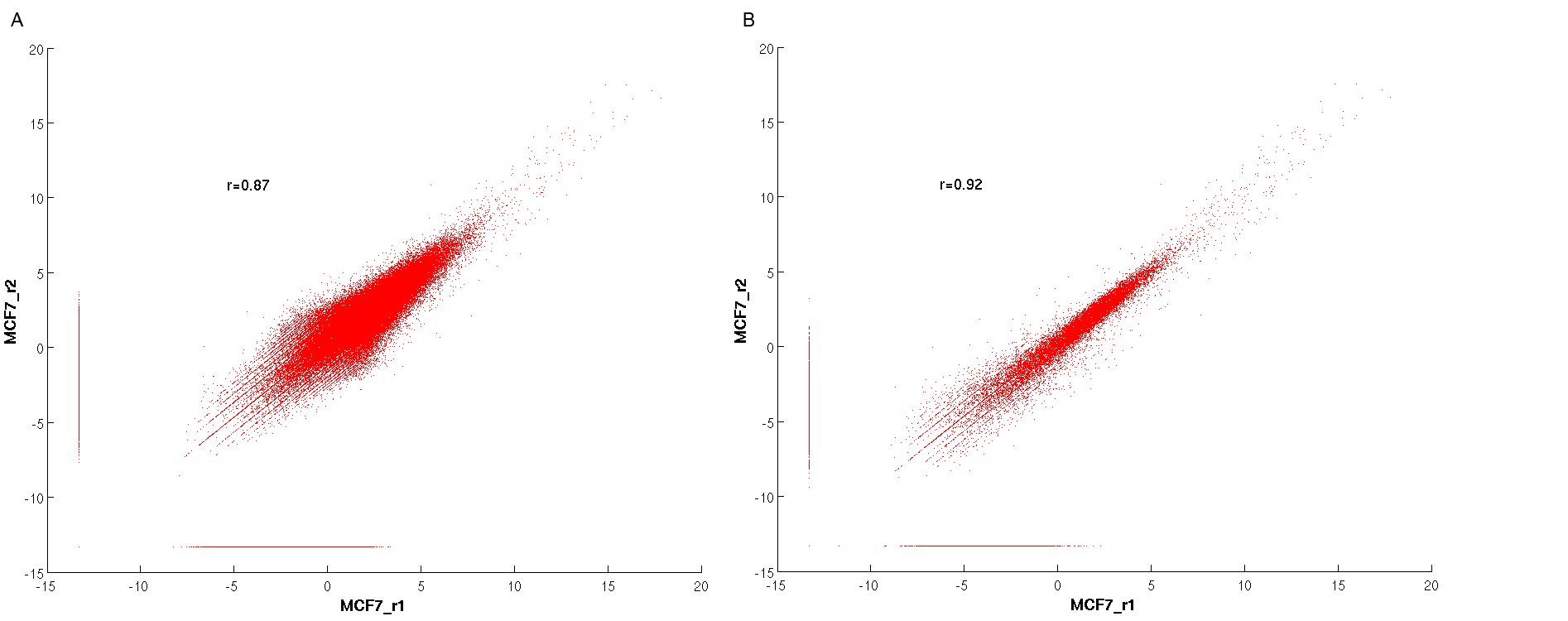


Supplementary Figure S1: Technical reproducibility of the RNA-Seq platform. The scatter plots show correspondence in (A) exon and (B) gene expression levels between MCF-7_r1 and MCF-7_r2.


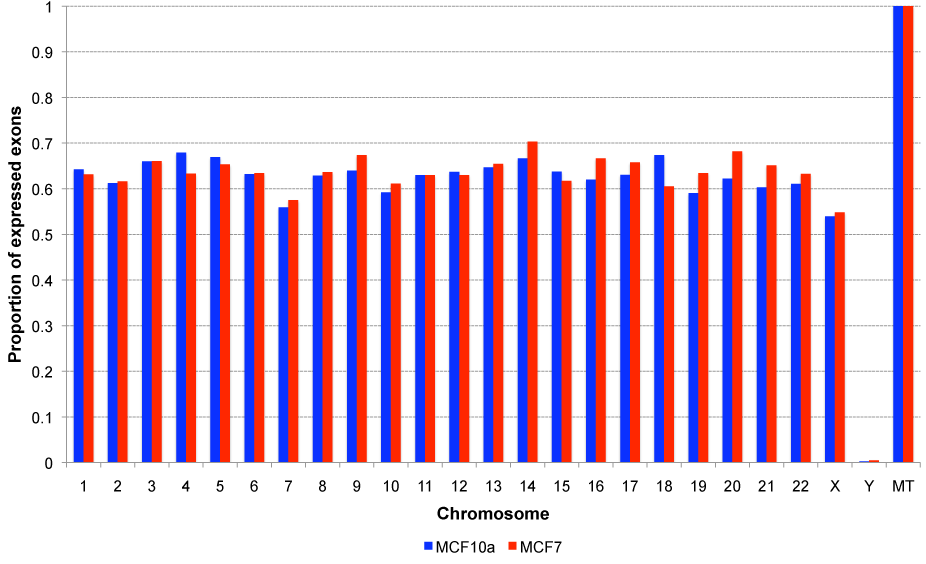


Supplementary Figure S2: The proportion of exons targeted by at least one read (“expressed exons”) across all 25 chromosomes in MCF-10a and MCF-7. Only exons whose length exceeded 100 bases after subtracting the number of non-unique loci (see Materials and Methods) were considered.


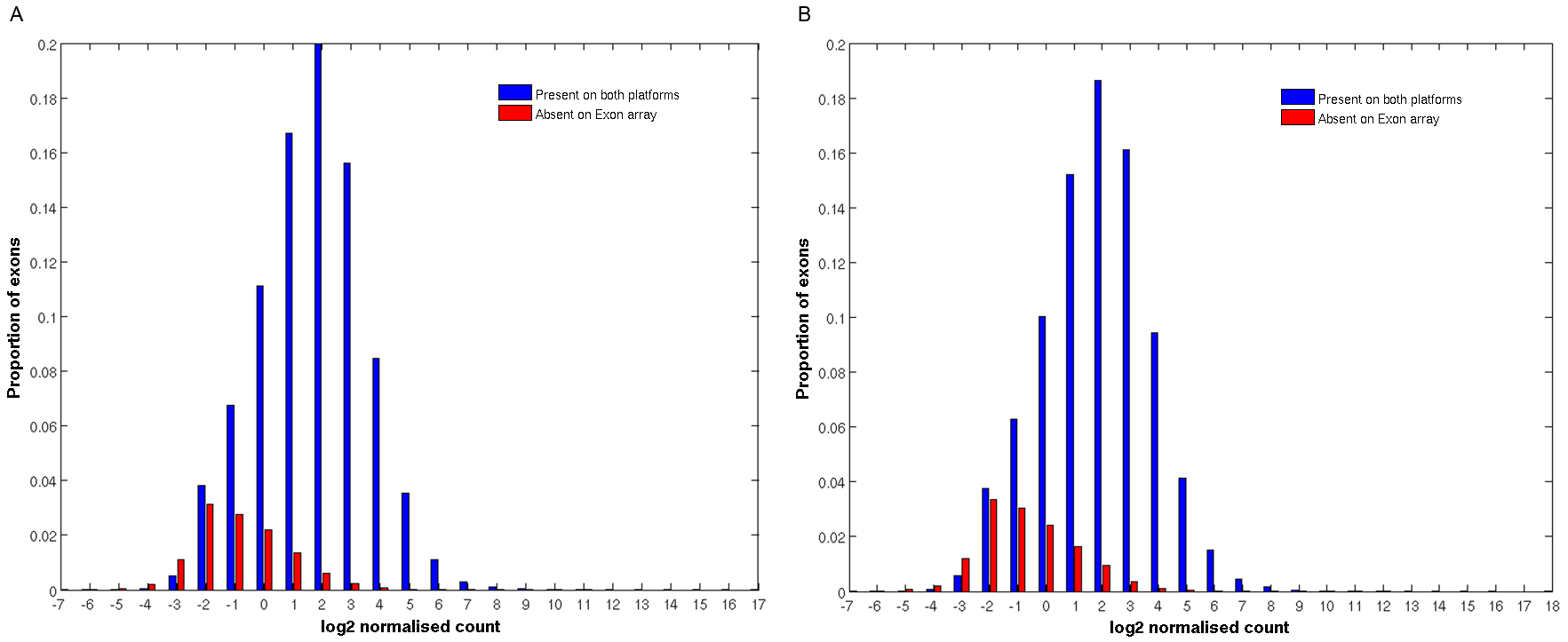


Supplementary Figure S3: Distribution of RNA-Seq expression levels in (A) MCF-10a and (B) MCF-7, of the 155,016 exons considered in the comparison with the Exon array. Blue bars represent exons Present on both platforms, and red bars indicate exons called Absent on the Exon array.


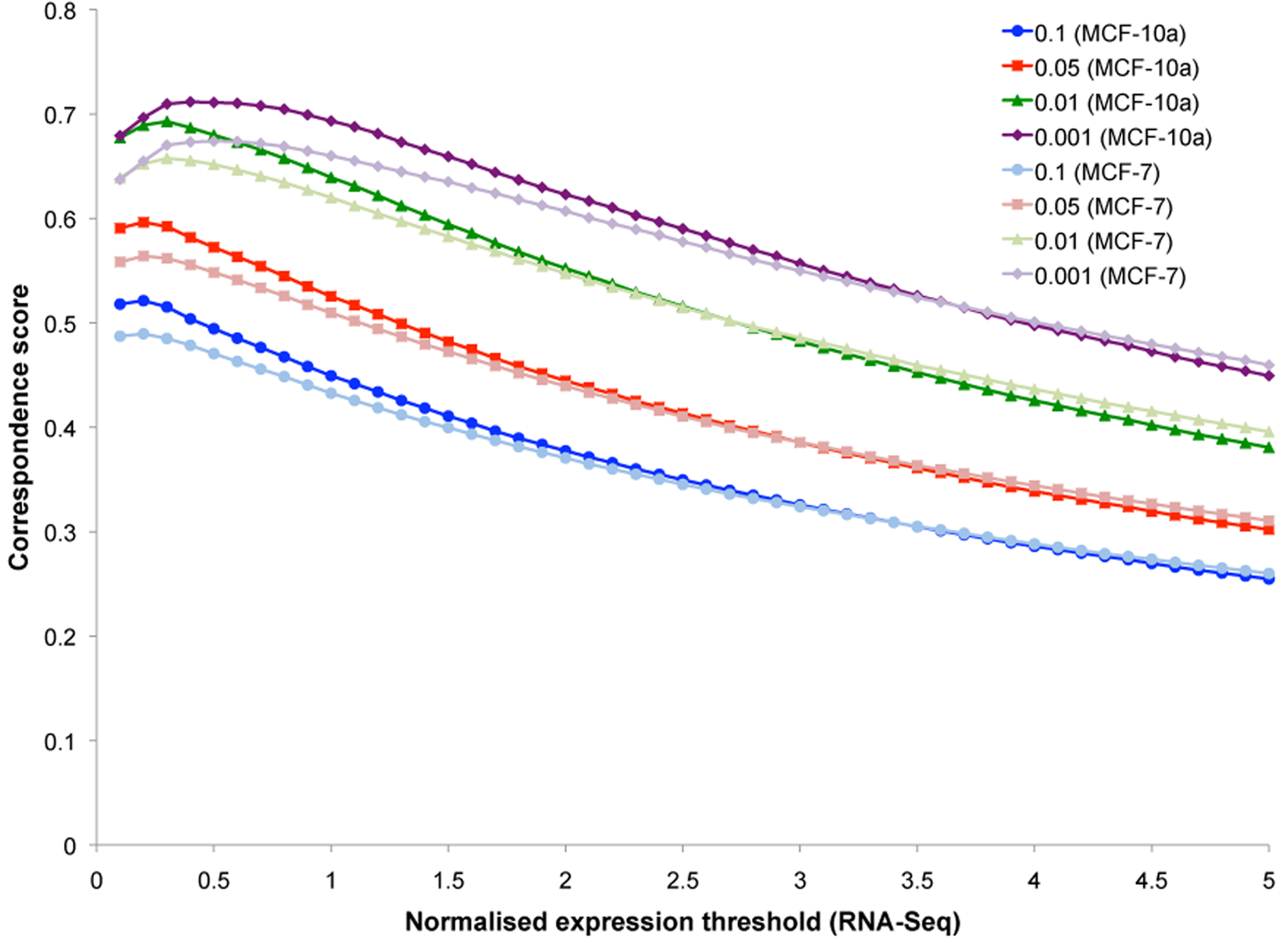


Supplementary Figure S4: Determining the read count threshold giving the optimum correspondence between the two platforms with respect to Present/Absent calls. Any exon above the read count threshold is defined as Present in the RNA-Seq data, while exon array probesets below a specified DABG p-score threshold were flagged Present. Four DABG score thresholds were considered. Only exons targeted by a single probeset are plotted.


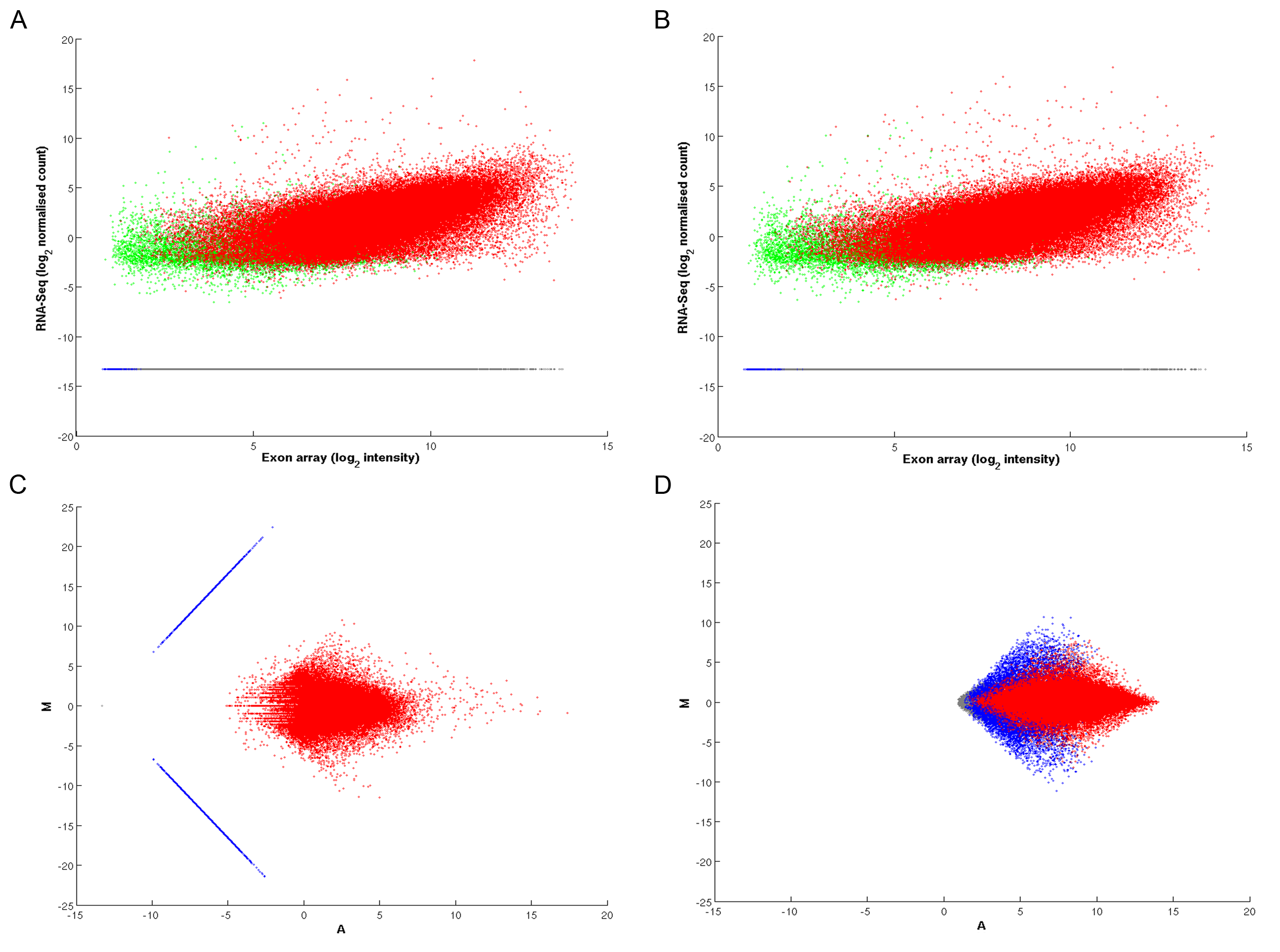


Supplementary Figure S5: Comparing normalised read counts across exons in RNA-Seq with probeset intensities from the Exon array in (A) MCF-10a, and (B) MCF-7. To avoid taking log of zero, we added 0.0001 to the normalised read counts. Red dots indicate exons with probesets flagged as Present on both SOLiD and the Exon array. Grey dots indicate exons with probesets flagged as Absent in RNA-Seq and the Exon array. Blue dots indicate exons with zero read counts but flagged Present on the Exon array, and green dots represent exons flagged Absent on the Exon array but with greater than one mappable read in RNA-Seq. (C) MA-like plot for the SOLiD platform (mean normalised read count across both cell lines versus fold change). Red dots indicate exons with an expression level greater than zero in both MCF-10a and MCF-7. Blue dots indicate exons with an expression level of zero in one cell line. Grey dots represent exons with a read count of zero in both cell lines. (D) MA plot for the Exon array. Red dots indicate exons flagged as Present in both MCF-10a and MCF-7. Blue dots indicate exons flagged as Absent in one cell line. Grey dots indicate exons flagged as Absent in both cell lines.


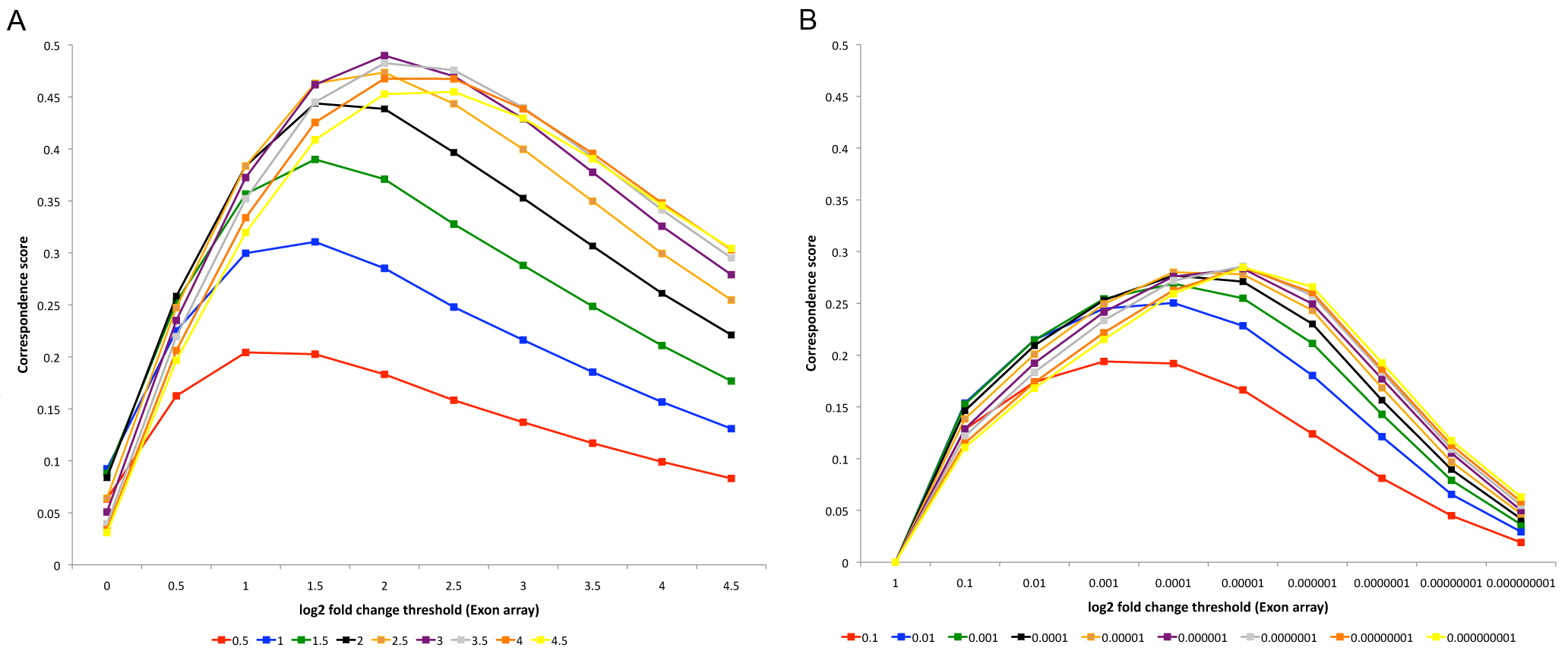


Supplementary Figure S6: Analysis of the set difference and intersection between exons called differentially expressed in RNA-Seq and on Exon arrays. Two methods to measure differential expression on SOLiD were used: fold change (A), and the Audic-Claverie (AC; B) statistical test, the detailed descriptions of which can be found in the methods. For all comparisons, triplicate Exon array data were used, and differential expression was found using LIMMA [4, 5]. The plots indicate the A. log_2_ fold change, and B. AC/LIMMA *p*-value thresholds that lead to the greatest equivalence between platforms using an overlap metric based on the *CS* (Equation 2). Legends indicate thresholds in RNA-Seq.


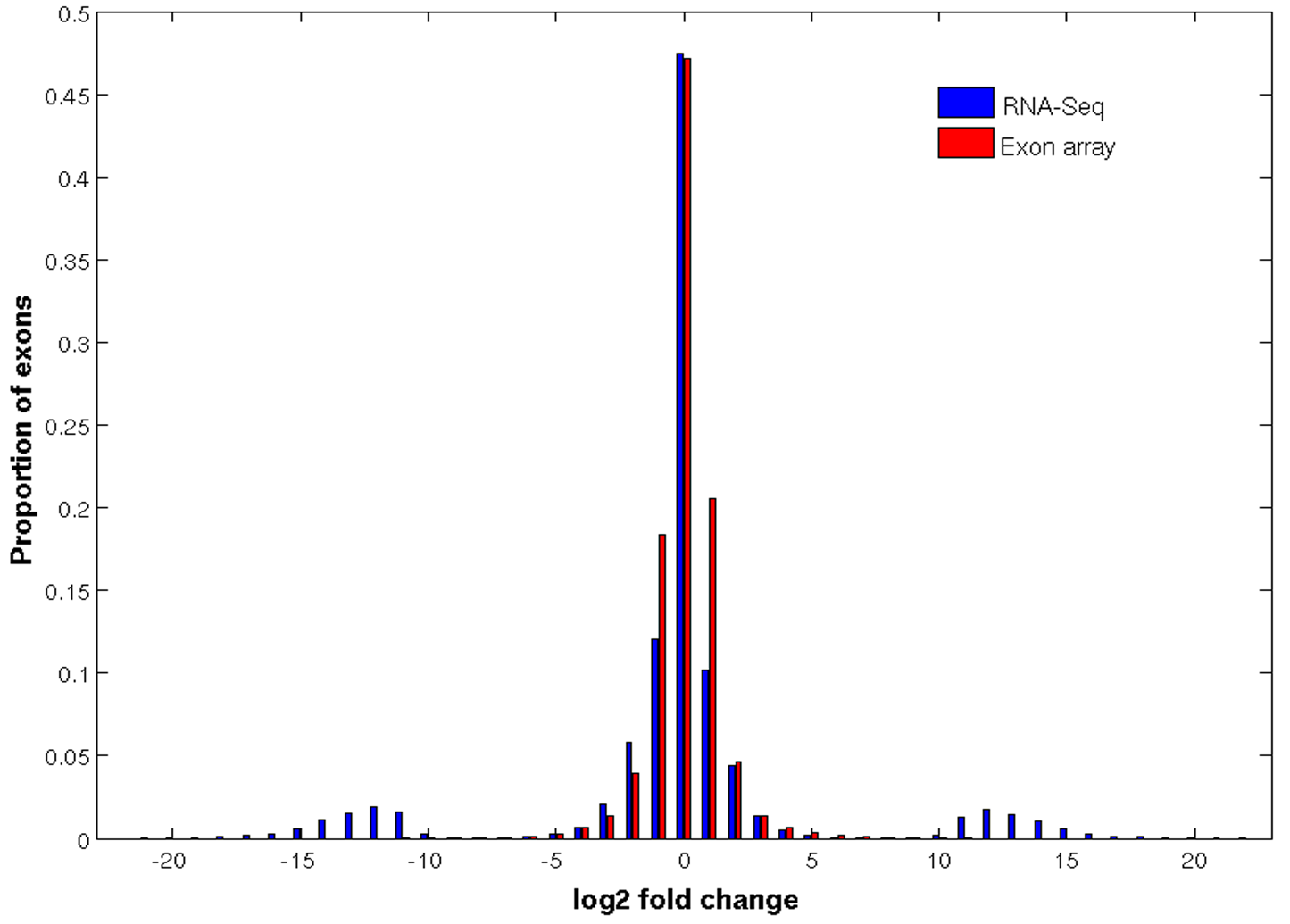


Supplementary Figure S7: Distribution of log_2_ fold change values between exons of MCF-10a and MCF-7. Blue and red bars represent the distribution in RNA-Seq and on the Exon array respectively.


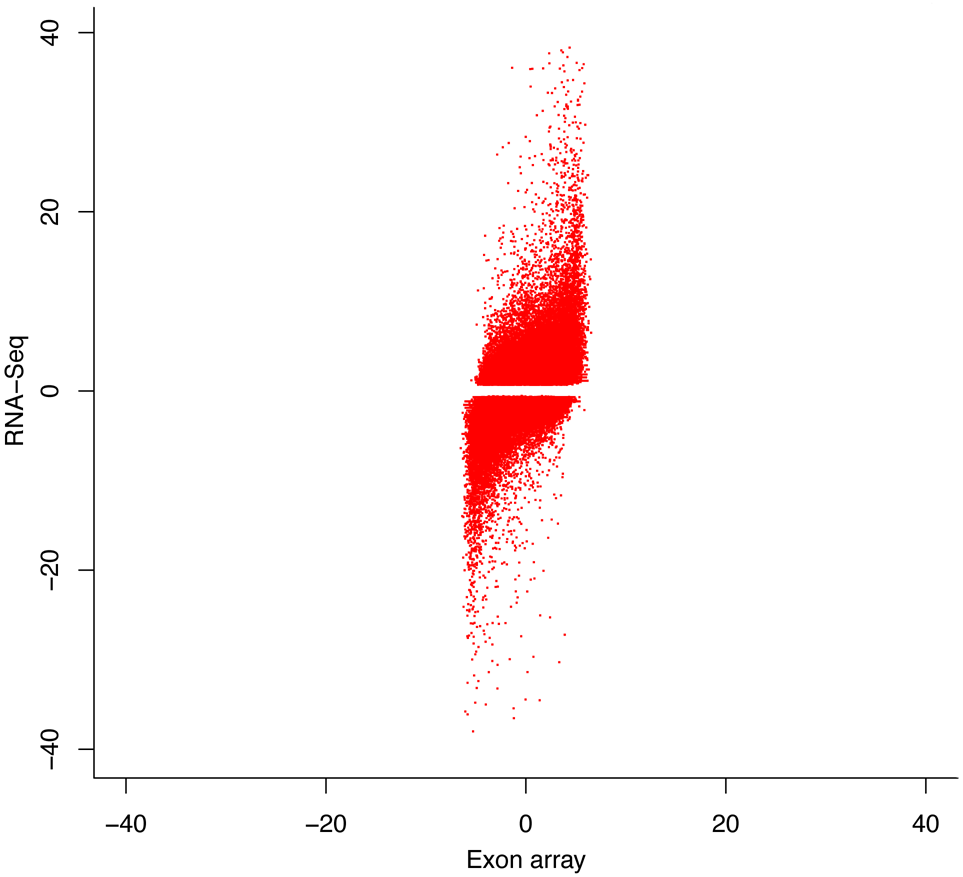


Supplementary Figure S8: The degree of correspondence (*r*=0.56) between the *p*-values calculated by LIMMA for the Exon array and AC for RNA-Seq. Values represent a back transformation of the *p*-values to quantiles on a normal distribution, signed by the direction of the fold change.


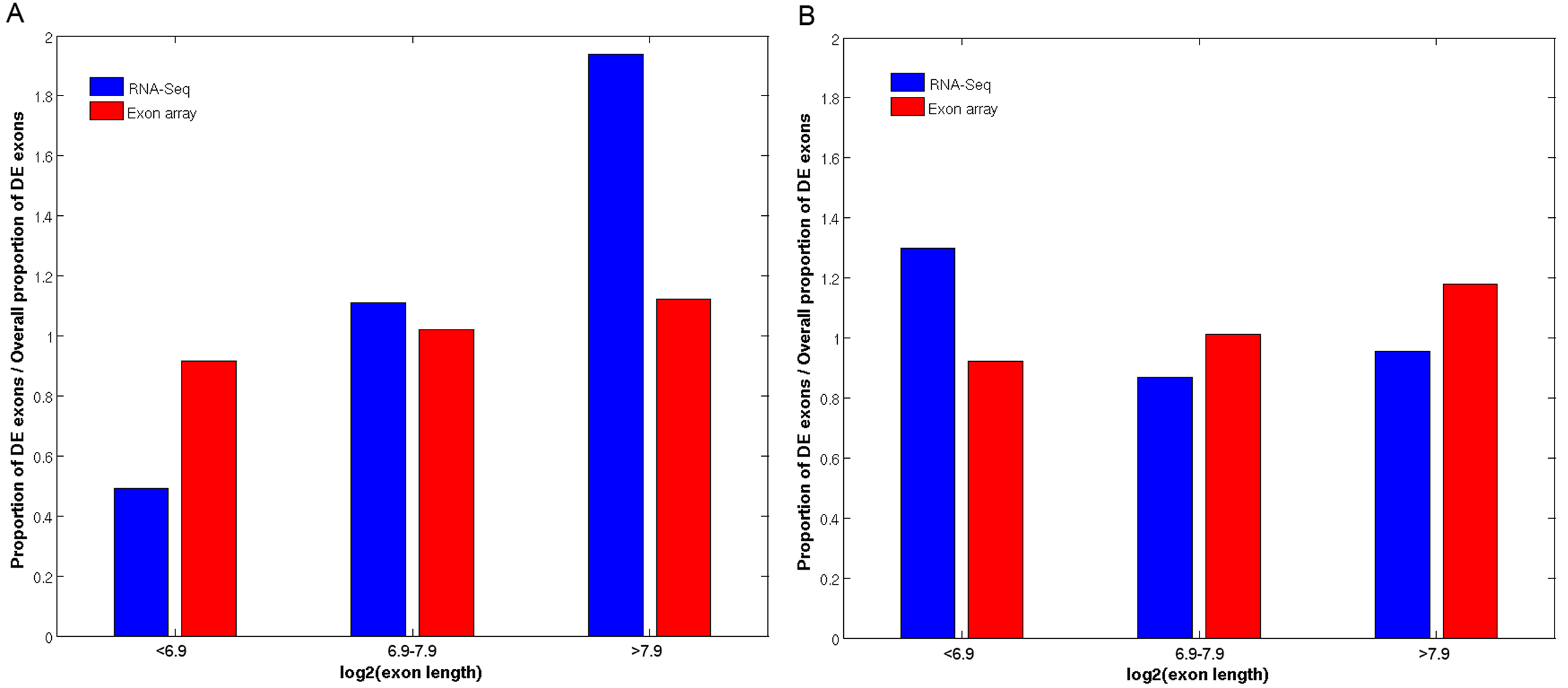


Supplementary Figure S9: Investigating the extent of length bias in calling differential expression on RNA-Seq using (A) *p*-values calculated by the Audic-Claverie (AC) measure (Equation 4), and (B) fold changes. Exon lengths were binned and the proportion of exons called differentially expressed in each bin calculated on both SOLiD (blue bars) and Exon array (red bars) based on the optimal fold change and *p*-value thresholds derived from Supplementary Figure 5. These were then normalised by the proportion of exons called differentially expressed across the whole dataset for each platform.


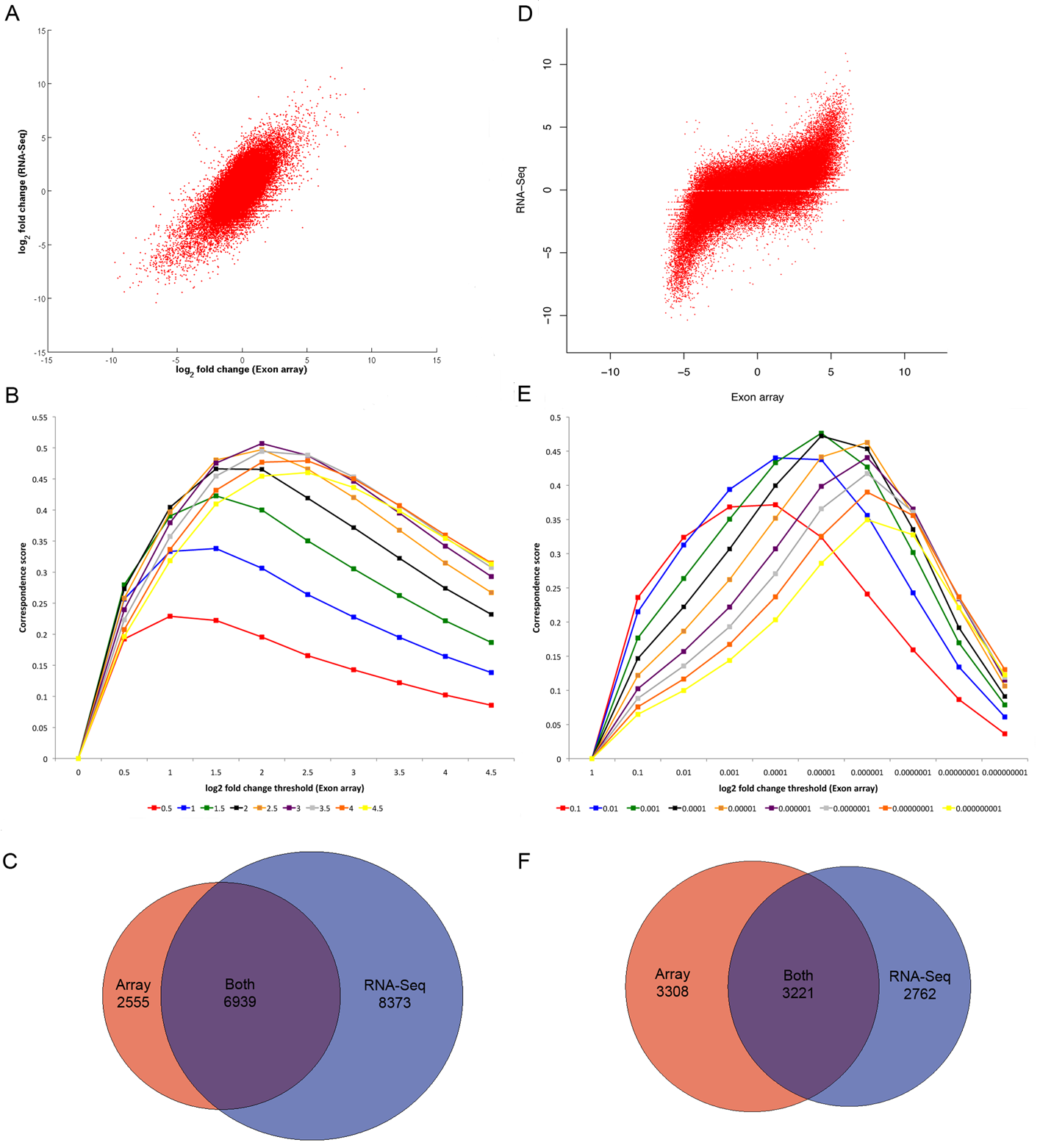


Supplementary Figure S10: Performance of differential expression measurements calculated by edgeR [6] in RNA-Seq , compared with those calculated by LIMMA [4, 5] on the Exon array. (A) Correspondence between fold changes (*r*=0.71). Only points corresponding to exons with read counts>0 in the MCF-10a sample and at least one of the MCF-7 replicates are shown. (B) Analysis of the set difference and intersection, indicated by *CS* (Equation 2), between exons called differentially expressed in RNA-Seq and on Exon arrays at various log_2_ fold change thresholds. (C) Overlap between exons called differentially expressed by the Exon array and RNA-Seq using a log_2_ fold change threshold of 2.0 on the Exon array and 3.0 on SOLiD. (D) The degree of correspondence (*r*=0.68) between the *p*-values. Values represent a back transformation of the *p*-values to quantiles on a normal distribution, signed by the direction of the fold change. (E) Equivalent to (B) but using *p*-value thresholds. Legends in (B) and (E) indicate thresholds using in RNA-Seq. (F) Equivalent to (C) but using an edgeR *p*-value threshold of 1x10^-3^ on the Exon array and 1x10^-5^ on SOLiD. These thresholds lead to the greatest equivalence between platforms using an overlap metric based on the *CS* (Equation 2).


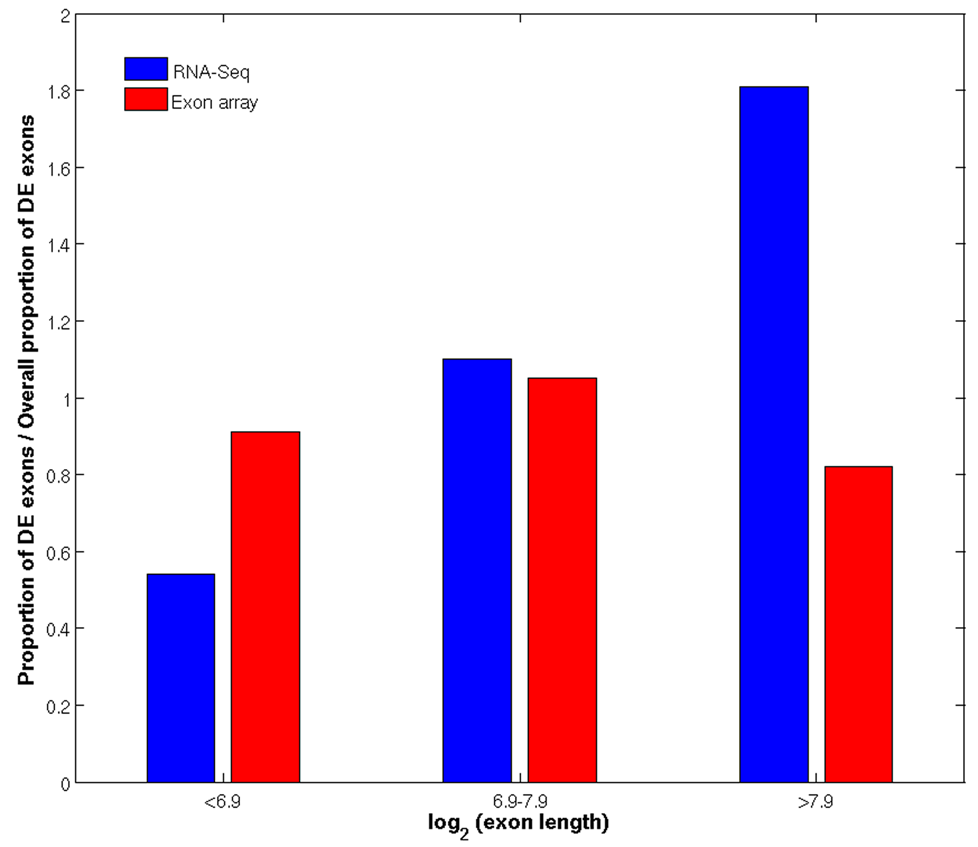


Supplementary Figure S11: Investigating the extent of length bias in calling differential expression on RNA-Seq using *p*-values calculated by edgeR. Exon lengths were binned and the proportion of exons called differentially expressed in each bin calculated on both SOLiD (blue bars) and Exon array (red bars) based on the optimal *p*-value thresholds derived from Supplementary Figure 9D. These were then normalised by the proportion of exons called differentially expressed across the whole dataset for each platform.


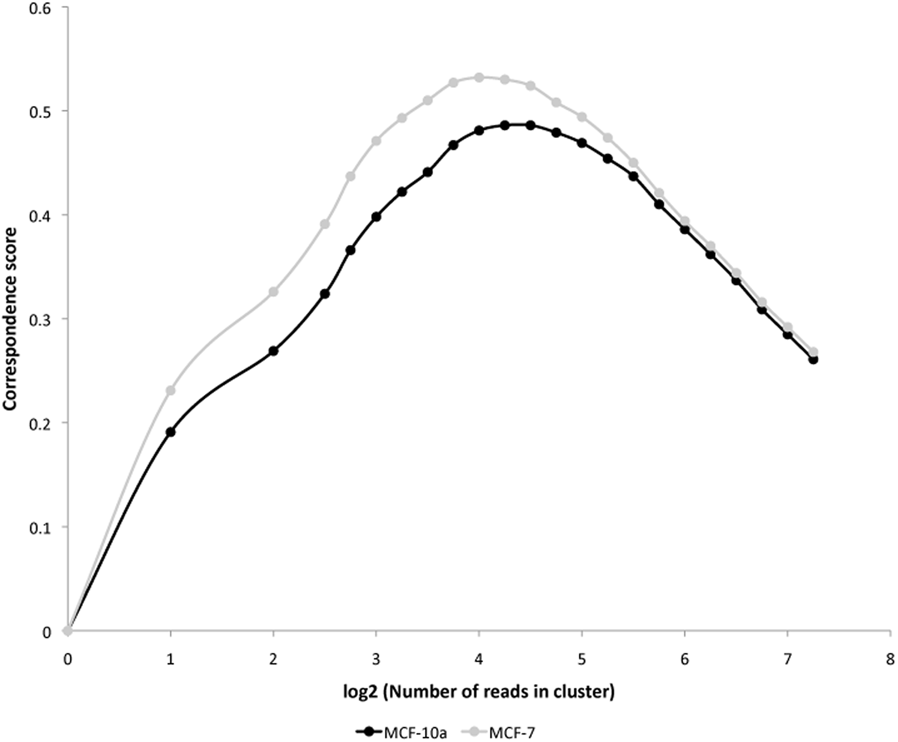


Supplementary Figure S12: Determination of a cut-off for calling novel exons. We used existing annotation to classify read clusters as exonic or intronic, and then defined a read count threshold that optimises the CS score in equation 2 (see Materials and Methods). The optimum threshold corresponded to a log_2_ read count of 4.25 (read count = 19) in MCF-10a and 4.0 (read count = 16) in MCF-7, at which exonic and intronic read cluster predictions most closely match the existing gene annotation.


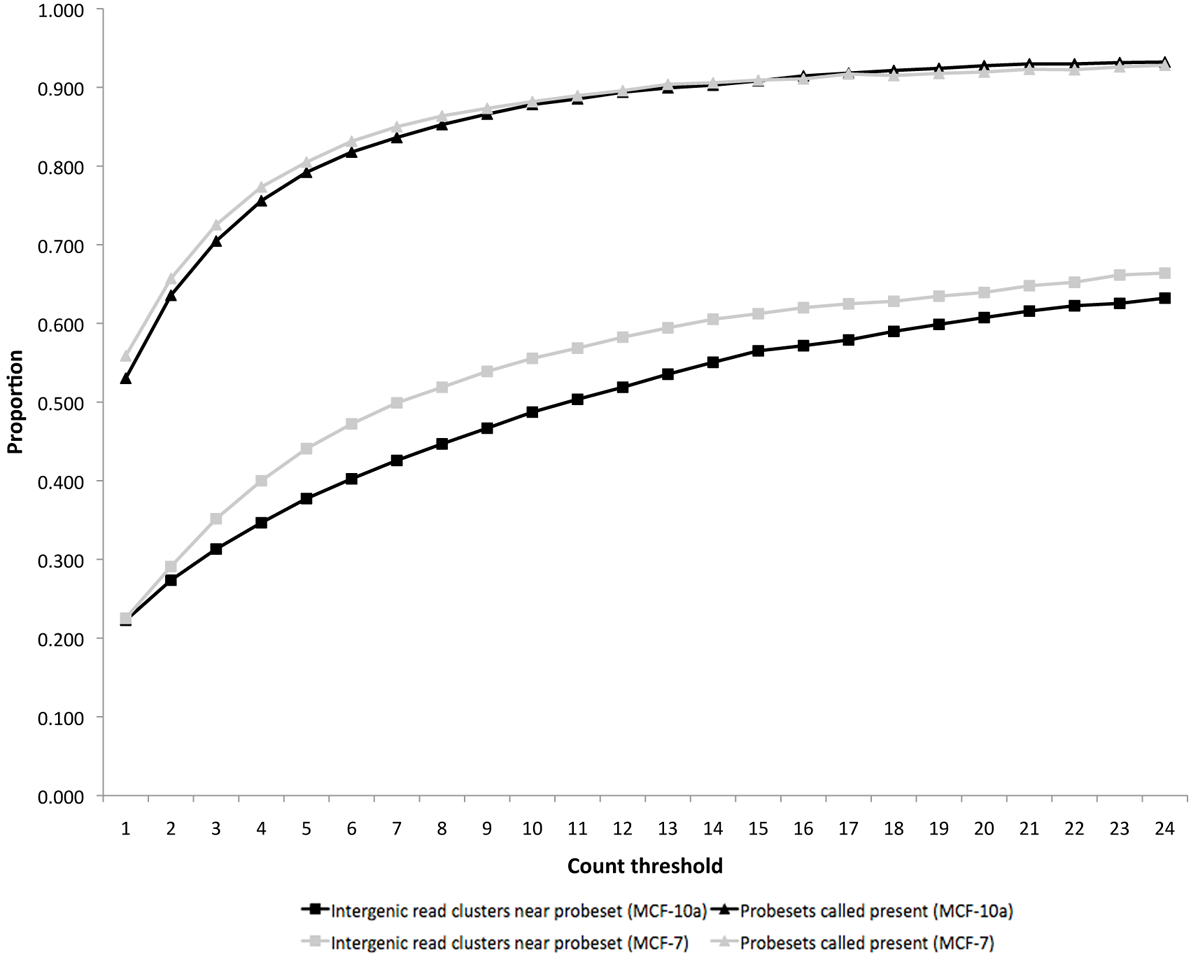


Supplementary Figure S13: Comparison between novel loci of transcription predicted from the RNA-Seq data, and the locations of non-core Exon Array probesets. The plots with square markers show how the proportion of read clusters located at or near a probeset increases with read count. The plots using triangular markers show the proportion of these neighbouring probesets called Present.


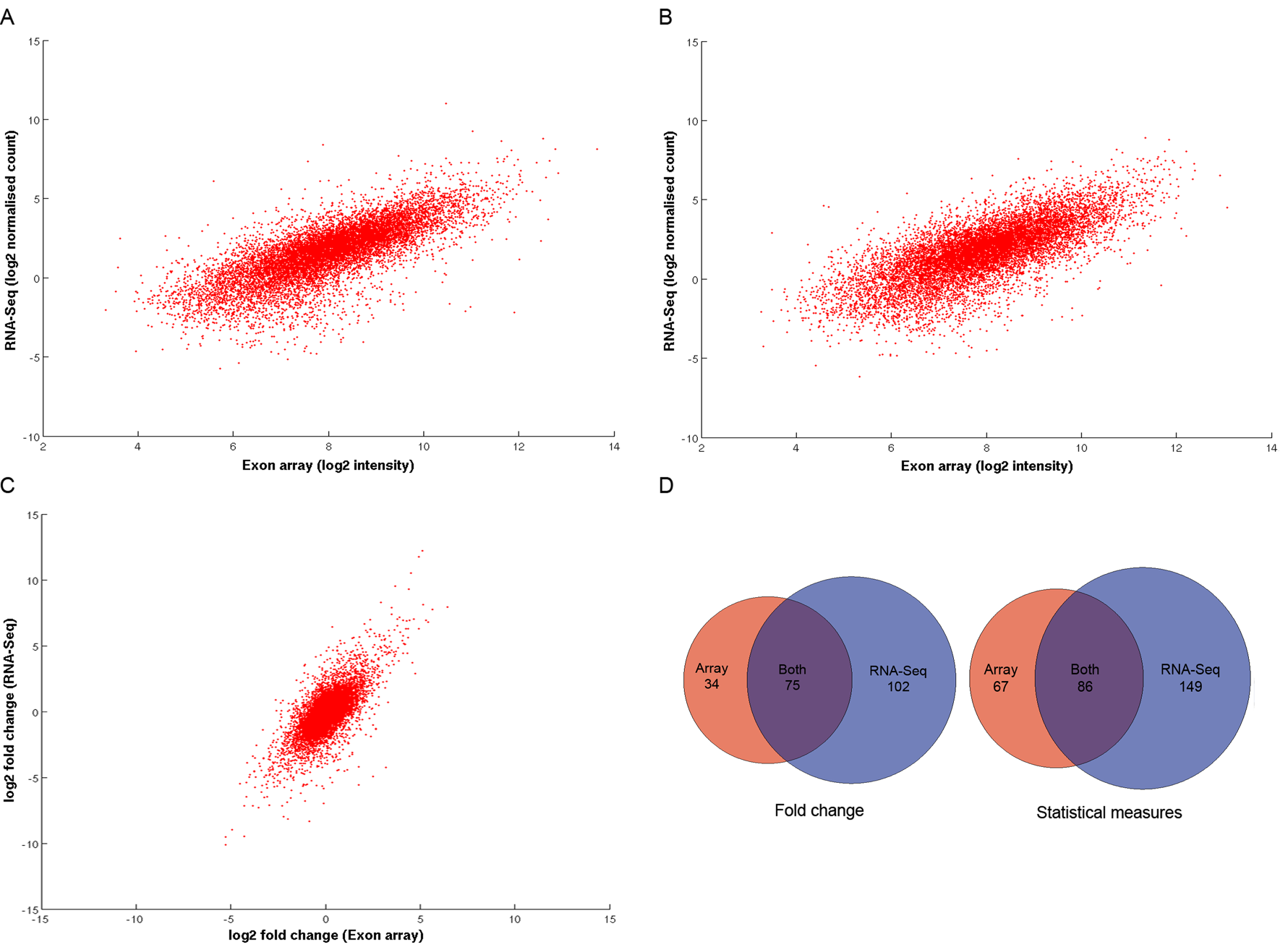


Supplementary Figure S14: Comparing normalised read counts across genes in RNA-Seq with probeset intensities from the Exon array in (A) MCF-10a, and (B) MCF-7. (C) Comparison of fold changes between genes from MCF-7 and MCF-10a calculated on each platform. (D) Venn diagrams summarizing the overlap between genes called as differentially expressed by the Exon array and RNA-Seq. The number of genes called by both technologies is indicated by the overlap between the two circles. The left Venn diagram shows the number of genes called differentially expressed using a log_2_ fold change threshold of 2.0 on the Exon array and 3.0 on SOLiD. The right Venn diagram is generated using Wilcoxon-ranksum *p*-value thresholds of 1x10^-4^ on both SOLiD and the Exon array. These thresholds lead to the greatest equivalence between platforms using an overlap metric based on the *CS* (Equation 2). In all figures, only genes comprising at least one exon with a read count above zero and a probeset called Present were considered.

References

1. Mortazavi A, Williams BA, McCue K, Schaeffer L, Wold B: **Mapping and quantifying mammalian transcriptomes by RNA-Seq**. *Nat Meth* 2008, **5**(7):621-628.

2. Marioni JC, Mason CE, Mane SM, Stephens M, Gilad Y: **RNA-seq: An assessment of technical reproducibility and comparison with gene expression arrays**. *Genome Research* 2008, **18**(9):1509-1517.

3. Finn RD, Tate J, Mistry J, Coggill PC, Sammut SJ, Hotz H-R, Ceric G, Forslund K, Eddy SR, Sonnhammer ELL *et al*: **The Pfam protein families database**. *Nucl Acids Res* 2008, **36**(suppl_1):D281-288.

4. Smyth GK: **Linear Models and Empirical Bayes Methods for Assessing Differential Expression in Microarray Experiments**. *Statistical Applications in Genetics and Molecular Biology* 2004, **3**:3.

5. Smyth GK, Michaud J, Scott HS: **Use of within-array replicate spots for assessing differential expression in microarray experiments**. *Bioinformatics* 2005, **21**(9):2067-2075.

6. Robinson MD, McCarthy DJ, Smyth GK: **edgeR: a Bioconductor package for differential expression analysis of digital gene expression data**. *Bioinformatics*, **26**(1):139-140.
